# Seeing the Middle: Reconstructing 3D Internal Electrode Microstructures from Low‐Resolution Surfaces with Generative Diffusion Artificial Intelligence

**DOI:** 10.1002/smsc.202500414

**Published:** 2025-09-23

**Authors:** Zhiqiang Niu, Zhaoxia Zhou, Patrice Perrenot, Claire Villevieille, Wanhui Zhao, Qiong Cai, Valerie J. Pinfield, Yun Wang

**Affiliations:** ^1^ Department of Aeronautical and Automotive Engineering Loughborough University Loughborough LE11 3TU UK; ^2^ Department of Materials Engineering Loughborough University Loughborough LE11 3TU UK; ^3^ Université Grenoble Alpes Grenoble INP LEPMI Université Savoie Mont Blanc CNRS Grenoble 38000 France; ^4^ School of Aero‐engine Shenyang Aerospace University Shenyang 110136 China; ^5^ School of Chemistry and Chemical Engineering University of Surrey Guildford GU2 7XH UK; ^6^ Department of Chemical Engineering Loughborough University Loughborough LE11 3TU UK; ^7^ Renewable Energy Resources Lab Department of Mechanical and Aerospace Engineering The University of California Irvine CA 92697 USA

**Keywords:** electrode microstructure reconstruction, fuel cells, generative artificial intelligence, multi‐physics modeling, solid‐state batteries

## Abstract

Characterizing the 3D complex energy materials interface is critical to understand the correlative relationship between performance, degradation, and structures. Unfortunately, the resolution of microscopy and image acquisition speed are limited by the nature of the hardware, causing high‐throughput characterization of energy materials to be prohibitive. Herein, REMind, a generative diffusion artificial intelligence model for fast and accurate reconstruction of electrode microstructures via focused ion beam‐scanning electron microscopy, is presented. REMind can generate high‐resolution internal microstructures between two low‐resolution surfaces after training on sufficient high‐resolution microstructures, enabling larger milling thickness between slices while keeping high‐fidelity imaging. REMind is first demonstrated for reconstructing solid oxide fuel cell (SOFC) anode microstructures. REMind resolves relevant multi‐scale structures with low pixel‐wise reconstruction error (<10%) and quantifies the generated uncertainty by calculating the generated entropy. Additionally, a multi‐scale multi‐physics SOFC model is employed to further quantify the reconstructed error regarding the electrochemical performance, i.e., operating current density versus overpotential. REMind shows good transferability, as proven by its ability to reconstruct other energy materials, including catalyst layers of proton exchange membrane fuel cells and solid‐state battery composite electrodes, demonstrating the potential for REMind to be used as a general‐purpose platform for broad development of energy technology.

## Graphical abstract

Herein, a generative diffusion artificial intelligence (AI) model, REMind, , is generated, which enhances energy material characterization by reconstructing high‐resolution electrode microstructures from low‐resolution slices. It enables faster, high‐fidelity imaging by allowing larger milling thickness in focused ion beam‐scanning electron microscopy. Demonstrated on solid oxide fuel cells, REMind achieves low reconstruction error and extends to other energy materials, accelerating energy technology development.

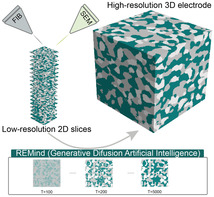

## 1 Introduction

Most physical, chemical, and materials scientists around the world often rely on advanced imaging techniques such as focused ion beam‐scanning electron microscope (FIB‐SEM) and X‐ray absorption tomography to gain insights into modern energy material sciences. These methods help to investigate whether perfect electrochemical interfaces are formed,^[^ ^1^, ^2^, ^3^ ^]^ how effective transport properties are correlated with electrode microstructures,^[^ ^4^, ^5^, ^6^ ^]^ or what micro/nanoscale defects cause quick performance degradation,^[^ ^7^, ^8^, ^9^ ^]^ etc. But large volume imaging via FIB‐SEM is always challenging, and time‐consuming, particularly when both high resolution and large volume are desirable. For FIB‐SEM, the full process of acquisition of a sample over days or weeks hinders high‐throughput screening and delays timely decision‐making on prototype samples. Imaging small volumes can lead to misinterpretations due to poor representativeness by excluding relevant features from the field of view.

The main effort in accelerating image acquisition via improving hardware focuses on exploring high‐energy beam resources and developing multi‐beam systems. For instance, emerging Xe^+^ Plasma FIB‐SEM achieved 60× material removal rate to traditional Ga^+^ FIB‐SEM because Xe^+^ beam can operate at high currents.^[^ ^10^, ^11^ ^]^ Moreover, multiple electron beams are designed to work in parallel to accelerate imaging speed, which makes acquiring an area of 1 mm^2^ at 4 nm resolution possible in only a few minutes.^[^ ^12^ ^]^ Additionally, cryogenic milling has been developed to reduce the potential damage from air and beam, and thus it allows higher beam currents to slice samples efficiently.^[^ ^13^ ^]^ In parallel to the efforts in improving FIB‐SEM, synchrotron X‐ray computed tomography employs extremely intense X‐ray beams to create high‐quality projections in a short time.^[^ ^14^, ^15^ ^]^ However, the aforementioned advances are made at the cost of increasing system complexity, potential beam damage that can bias the obtained results and increase costs, preventing them from becoming widely accessible to broader users.

Following the rise of deep learning,^[^ ^16^, ^17^, ^18^, ^19^, ^20^, ^21^ ^]^ rapid advances in data reconstruction are poised to open a new frontier for cost‐effective high‐resolution FIB‐SEM in energy science and engineering.^[^ ^22^, ^23^, ^24^, ^25^, ^26^, ^27^, ^28^, ^29^, ^30^, ^31^ ^]^ Super‐resolution tasks with deep learning have been achieved to enable a large field of view with high resolution.^[^ ^25^, ^30^ ^]^ Various high‐resolution energy material structures including nanoparticles,^[^ ^25^ ^]^ battery electrodes^[^ ^29^ ^]^ and fuel cell electrodes^[^ ^30^ ^]^ have been reconstructed from low‐resolution slices by multiple super‐resolution models, such as super‐resolution (SR) convolutional neural networks and SR generative adversarial neural networks(SRGANs). For instance, Kench and Cooper^[^ ^17^, ^18^ ^]^ developed a bespoke GAN model that can generate 3D microstructures from 2D slices. Their SliceGAN model was developed by taking a single 2D slice and then generating arbitrarily large 3D microstructures. They further transferred the GAN model to generate high‐resolution electrode microstructures through the fusion of 2D high‐resolution and 3D low‐resolution data.^[^ ^30^ ^]^ Both SliceGAN and data diffusion generative models have significantly advanced the efficiency and the quality of generated 3D microstructures. Recent breakthroughs in denoised diffusion probabilistic models have opened new opportunities for generating high‐quality samples with bespoke electrode thickness and a stable training process.^[^ ^31^ ^]^ However, limited efforts have been made to explore how generative diffusion models can be customized for both in‐plane and through‐plane super‐resolution, particularly in the context of FIB‐SEM. Moreover, more diverse criteria, e.g., the electrochemical performance, are needed to assess the reconstruction quality of porous electrode microstructures beyond mean squared error^[^ ^32^ ^]^ or perceptual similarity.^[^ ^33^ ^]^ Yet, quantifying the electrochemical performance of digital material structures is imperative to make decisions on the material fabrication and optimization.^[^ ^34^, ^35^, ^36^, ^37^ ^]^

Here, we present a deep learning framework, REMind, which can reconstruct internal electrode microstructures between two low‐resolution slices along the milling direction via a conditional generative diffusion model. REMind achieves both in‐plane and through‐plane super resolutions and thus accelerates the milling processes and enlarges the field of view, allowing the reconstruction of large digital samples at low cost and high resolution. The stable training of REMind is enabled by a conditional denoised diffusion probabilistic model, a type of generative artificial intelligence that can model the probability of complex data and generate new samples. Coupled with multi‐scale physical models, the electrochemical performance, i.e., overpotential versus current density curves, serves as an additional indicator to assess the reconstruction quality. We demonstrated the effectiveness of REMind on various electrochemical material structures, including fuel cell electrodes and solid‐state lithium‐ion battery (SSB) composite electrode structures, providing a cost‐effective approach towards high‐throughput FIB‐SEM image acquisition.

## 2 REMind

**Figure 1:**
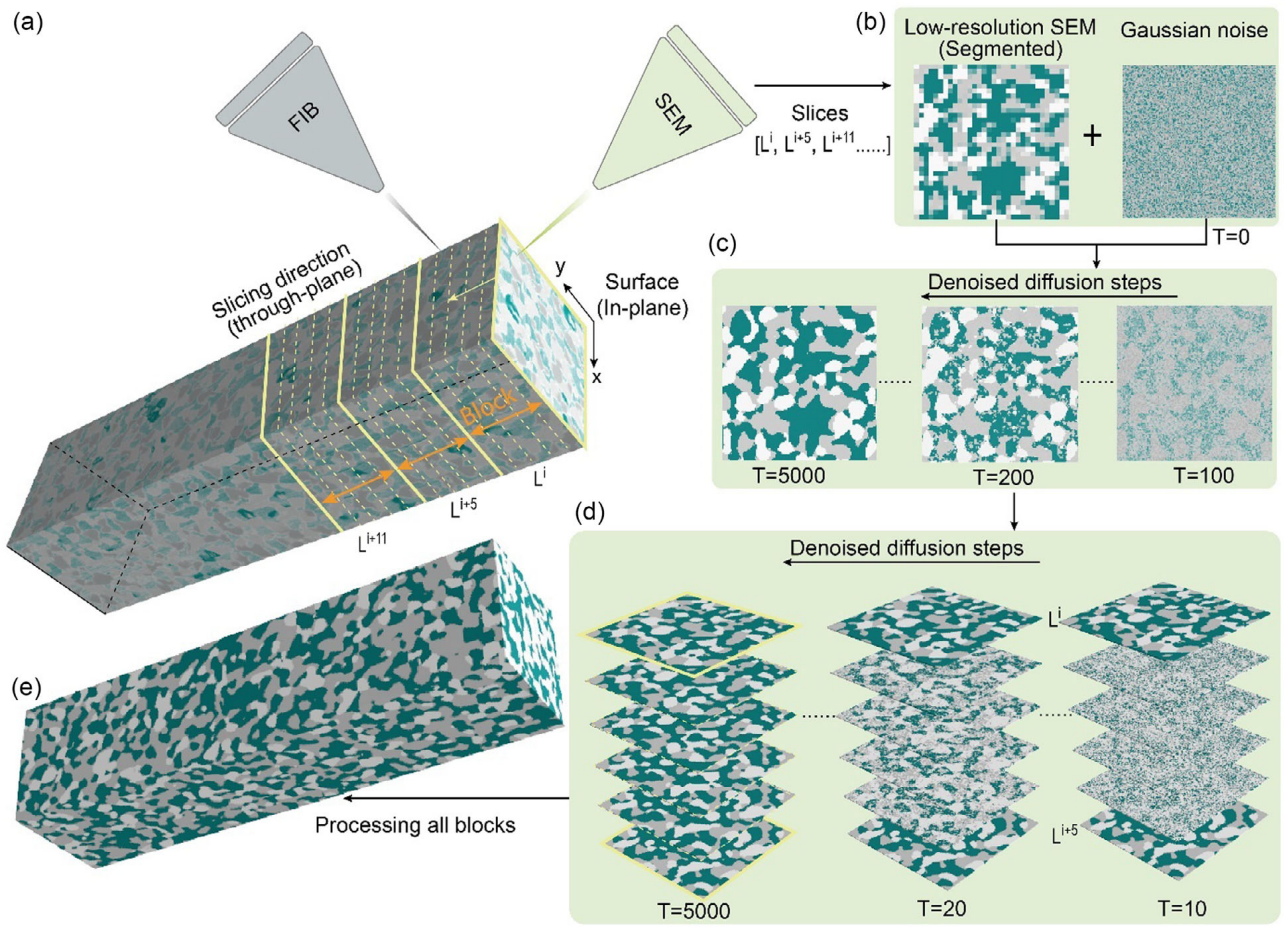
Schematic of REMind reconstructing internal microstructures from low‐resolution surface data. a) A schematic of FIB‐SEM and how blocks are sliced and imaged by FIB and SEM, respectively. b) Low‐resolution SEM images of the block surfaces (the slices highlighted by the solid yellow line) alongside a Gaussian noise as input for REMind. c) In‐plane super‐resolution by the generative diffusion model, which iteratively denoises the Gaussian noise conditioned on the low‐resolution surfaces. d) Through‐plane reconstruction of internal microstructures (the slices highlighted by yellow dashed lines) of a given block. e) The ultimate digital structures of an SOFC electrode assembled by a series of connected blocks.

**Figure** **1** depicts how REMind cooperates with FIB‐SEM to sample solid oxide fuel cell (SOFC) electrode microstructures. The first stage is for the in‐plane resolution, where REMind takes low‐resolution surface SEM images of slicing blocks as input and subsequently outputs high‐resolution block surface structures. This is achieved by a conditional generative diffusion model, which iteratively refines a field of initial Gaussian noise, conditioned on the low‐resolution surface SEM images, as shown in Figure 1a–c. Notably, 2D high‐resolution slices are necessary for the model super‐resolution training before REMind can generate high‐resolution slices from low‐resolution input. The similar use of 2D high‐resolution slices for super‐resolution training was also employed in a previous study.^[^ ^30^ ^]^ The second stage is for the through‐plane reconstruction, where REMind reconstructs the internal structures in each block by only taking the top and bottom low‐resolution surface as input, see Figure 1d. Notably, the reconstruction is implemented by the previous conditional generative diffusion model with different conditional inputs. The ultimate digital structure is obtained by REMind after processing all blocks, as shown in Figure 1e. Further details of the denoised diffusion process are presented in the Experimental section.

## 3 Results and Discussion

### 3.1 In‐Plane Surface Super‐Resolution

**Figure 2:**
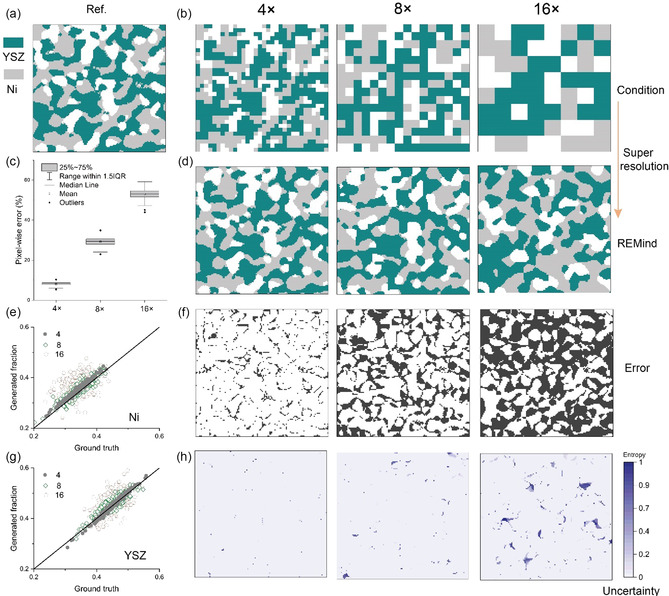
The performance of REMind in achieving in‐plane super‐resolution for the SOFC anode. a) High‐resolution image (128^2^ voxels) used as ground truth. b) Low‐resolution images provided as input conditions for REMind under three super‐resolution factors: 4×, 8×, and 16×. c) Statistics of the pixel‐wise errors. d) Super‐resolution in‐plane surfaces generated by REMind. e) Error distribution of the generated volume fraction of Ni across 200 samples. f) Spatial error distributions. g) Error distribution of the generated volume fraction of YSZ across 200 samples. h) Entropy maps for uncertainty quantification.

We tested the super‐resolution accuracy of REMind on the in‐plane surface under varying super‐resolution factors, i.e. 4×, 8×, and 16×. A higher super‐resolution factor indicates a lower‐resolution 2D slice is taken as the conditional input by REMind. Super‐resolution factor also indicates the ratio between the output and input size of REMind. The super resolution ability of REMind is currently designed for tasks with even factors such as 4× and 8×. However, the parameters of convolutional layers, such as padding, can be tuned to adapt REMind for diverse scale factors. The model was tested on a segmented SOFC anode dataset. REMind generated respective super‐resolution surfaces in **Figure** **2**d by taking three types of low‐resolution images (32^2^ pixels for 4×, 16^2^ pixels for 8× and 8^2^ pixels for 16×) in Figure 2b as conditions. Notably, the physical size (8.32^2^ μm^2^) of the region of interest remains the same for three cases. The 4× case outperforms as its has a high similarity as the ground truth which was obtained by using 128^2^ voxels to resolve the region of interest, as shown in Figure 2a. The 8× case partially reconstructs large‐scale features but is relatively poor in recovering small‐scale features and the connectivity of yttria‐stabilized zirconia (YSZ). Although the 16× case contains some electrode features, they mismatch the ground truth. The corresponding spatial error distributions are presented in Figure 2f where black spots mark the error locations. The reason for the increasing super‐resolution error is that the condition from the case of high super‐resolution factors lacks multi‐scale details to guide REMind towards accurate local feature reconstruction.

Figure 2c further shows the pixel‐wise error for 200 test samples under three factors. The averaged super‐resolution error is observed to increase almost linearly from 8.08% to 53.14%. To quantify the super‐resolution error of individual components of the SOFC electrode, Figure 2g,h compares the generated volume fraction and ground truth for nickel (Ni) and YSZ, respectively. It is observed that both comparisons exhibit similar scattering; however, the super‐resolution of YSZ is lower than that of Ni. This discrepancy is likely attributed to the higher volume fraction of YSZ. Super‐resolution is widely recognized as a naturally ill‐posed problem with multiple solutions. Uncertainty quantification plays an important role in the trust of material scientists in the output of REMind and is given in Figure 2h for the super‐resolution outputs by using entropy, which ranges from 0 to 1. Each entropy map was prepared by the statistical analysis of 200 outputs for a given input (see Experimental Section for details). As seen in Figure 2h, the majority area in case 4× exhibits significantly low uncertainty, with only a few sparse spots of high uncertainty. In contrast, case 16× reveals extensive regions characterized by high uncertainty. Thus, 4× was chosen for subsequent through‐plane reconstruction in the study.

### 3.2 Through‐Plane Reconstruction

**Figure 3:**
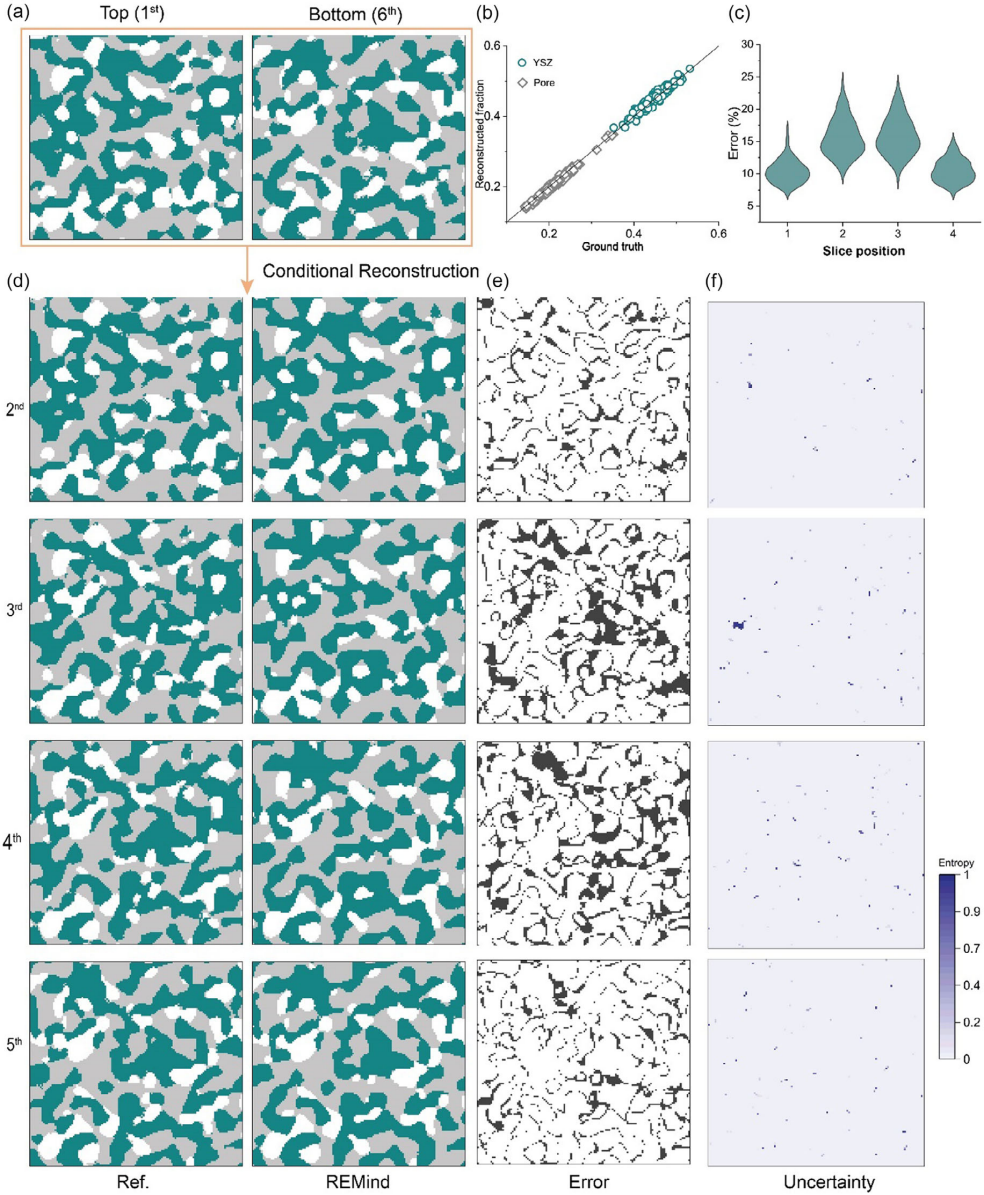
REMind performance for through‐plane internal microstructure reconstruction of the SOFC anode. a) The top and bottom slices of a given block are provided as conditional input. b) Distribution of the generated volume fraction of pore and YSZ across 200 samples. c) Spatial averaged error distributions along the through‐plane direction. d) Ground truth and reconstructed internal microstructures of the given block conditioned on the surface information in (a). e) Spatial error distributions along the through‐plane direction. f) Entropy map for uncertainty quantification.

Traditional high‐resolution FIB‐SEM imaging requires a very fine milling distance along the through‐plane direction. Increasing the milling distance between slices (see Figure 1a) is an effective solution to accelerating FIB‐SEM acquisition; however, it requires advanced digital algorithms to reconstruct internal microstructures between two distinct surfaces. We demonstrate that REMind successfully reconstructed internal block microstructures by using the in‐plane super‐resolution surfaces in Section 3.1 as input conditions. **Figure** **3**a,d shows how REMind reconstructs the internal microstructures for a block consisting of 6 slices by taking its top (1^st^ slice) and bottom (6^th^ slice) as input conditions. Overall, the reconstructed error is acceptable, as evidenced by the volume fraction of pore and YSZ in Figure 2b. However, the spatial error varies along the through‐plane positions. For instance, the reconstructed slices (e.g., slices 2^nd^ and 4^th^ near the top and bottom) show lower error compared to the reconstructed middle slices, as shown in Figure 3c. This is because the convolution layers in REMind have limited receptive fields and tend to capture weak correlation between the conditional surface and the middle surfaces. The detailed spatial error distributions are presented in Figure 3e. The overall uncertainty is low except for a few areas in the middle slices, as seen in Figure 3f.

Next, we investigated how the reconstruction accuracy varies with the thickness of a given block. Here, the thickness *L* of a given block is defined by the number of slices, each separated by a fixed distance increment of 65 nm. A set of *L* values, i.e., 4, 8, 10, and 12,was further studied. Figure S2, Supporting Information, shows a base case *L* = 4 for comparison. Figure S1b,c, Supporting Information depicts significantly low reconstructed error in terms of reconstructed volume fractions and local errors (<10%) along the through‐plane direction, as well as low uncertainty across the whole region. However, the reconstructed error increases as the slice number is further increased, especially the reconstructed error for middle slices in cases of *L* = 8, 10, and 12, as shown in Figure S2–S6, Supporting Information. For case *L* = 8 in Figure S2, Supporting Information, the averaged error at the two middle slices is around 25%, while it exceeds 50% for case *L* = 12, indicating the limited ability of REMind to reconstruct thicker blocks. Meanwhile, the uncertainty (Figure S6b, Supporting Information) is high around the middle slices under large *L*, further altering the application of REMind in super‐large slice numbers. It is observed from Figure 3 and S1–S5, Supporting Information that the accuracy of through‐plane reconstruction depends on the choice of *L*. Two key factors should be considered when selecting *L*. First, the characteristic feature size of the material, particularly in heterogeneous porous energy materials, is critical. Ideally, the physical length corresponding to *L* should match the characteristic feature size (*L* = 6 recommended for SOFC electrode). For instance, the characteristic grain size in the real SOFC sample is around 400–800 nm, as shown in Figure S7d, Supporting Information, indicating that L needs to be around 6 to capture relevant grain features when the pixel resolution is 65 nm. The L tends to be low when the microstructures contain fine structures as a characteristic feature. Second, the receptive field of the convolutional neural network used in REMind should be considered. The receptive field increases with the number of convolutional layers, allowing the network to capture long‐range correlations between pixels along the through‐plane direction; however, increasing the number of layers also raises the computational burden. Notably, the characteristic feature size can be technically approximated by using autocorrelation or power spectral density analysis, which can evaluate particle size distribution. These algorithms have been implemented in Image J Fiji and PoreSpy, an open‐source quantitative image analysis of porous materials.^[^ ^38^ ^]^ Additionally, TauFactor is also helpful to identify a representative region of interest through the calculation of effective transport properties.^[^ ^39^ ^]^

**Figure 4:**
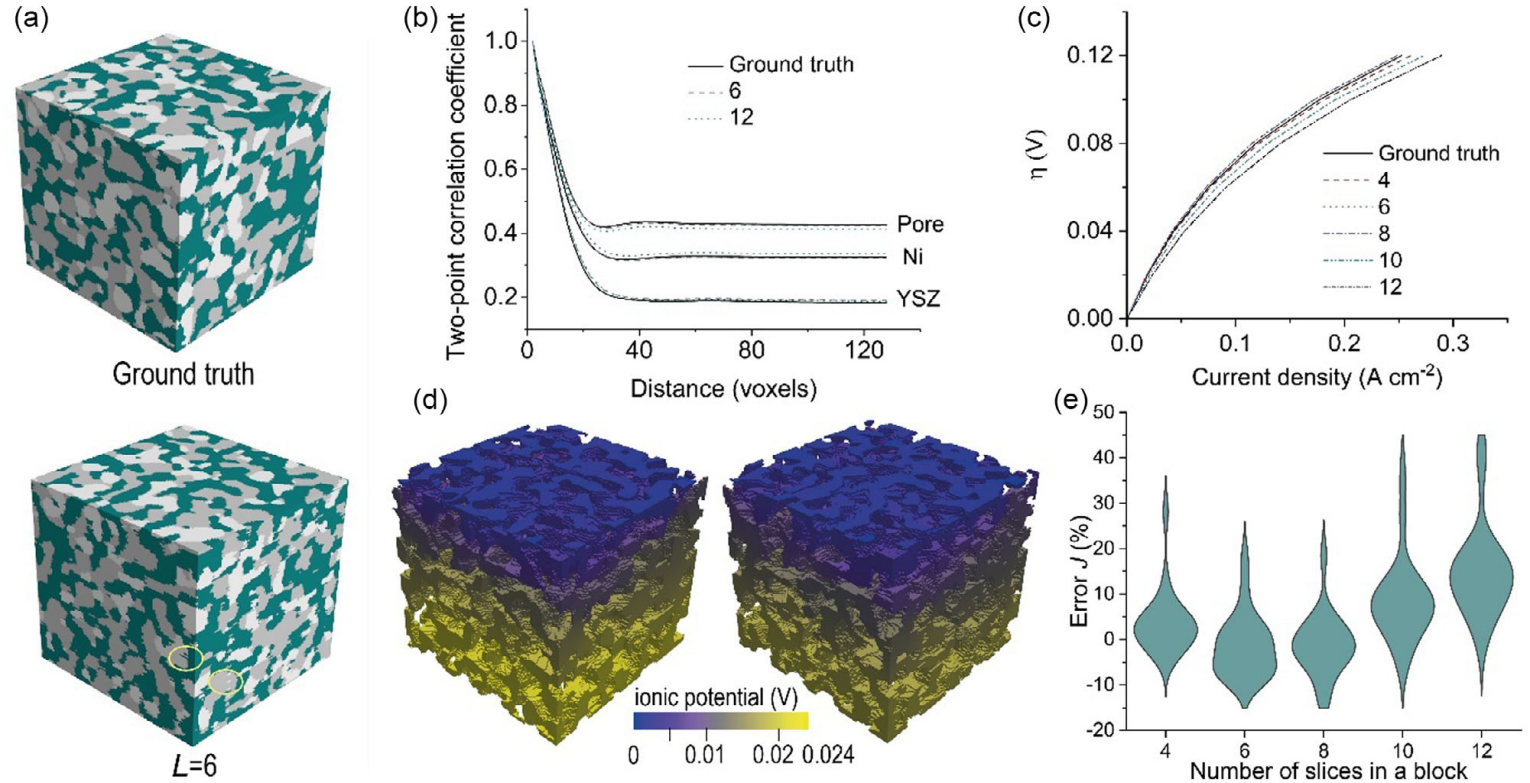
Structural and electrochemical evaluation of the reconstructed SOFC anode by REMind. a) Two samples reconstructed by an Xe FIB‐SEM (ground truth) and REMind (*L* = 6), and the discontinuous stripes are highlighted by the yellow circle. b) Two‐point correlation coefficients averaged across 20 samples. c) Averaged *J*‐*η* curves across 20 samples under *L* = 4–12. d) Distributions of ionic potential for two samples (Left: ground truth; right: *L* = 6). The difference between the two distributions can be identified by the dark light yellow (left) and dark yellow (right) in the lower regions. e) The local error distribution of *J* under *η* = 0.12 V for cases *L* = 4–12.

The ultimate FIB‐SEM sample is obtained by stacking a series of slices, as shown in **Figure** **4**a. The total times taken by REMind to reconstruct a single 120 × 128 × 128 voxel sample are 20, 24, 29, 34, and 40 mins for cases 4–12, respectively. It is seen that the SOFC electrode from REMind resembles the sample consisting of 120 slices reconstructed by the traditional FIB‐SEM. However, some elongated features highlighted in the reconstruction by REMind mismatch the ground truth. Although the error analysis was conducted for individual blocks, the error remains unclear for the whole reconstructed sample. To further assess the properties of the ultimately reconstructed sample, we not only conducted a structural analysis including two‐point correlation, specific area density, tortuosity, and triple‐phase boundary (TPB) but also employed a multi‐physics, multi‐scale SOFC electrode model to reveal the reconstructed *J*‐*η* performance.

Figure 4b depicts the two‐point correlation coefficients for three components of the SOFC anode, i.e., pore, Ni, and YSZ. The good agreement between the two‐point correlation curves indicates that both samples are nearly identical in terms of their structural properties, even when a large *L* was used. The comparison of specific surface density (pore/YSZ, pore/Ni, and Ni/YSZ), tortuosity of the pore phase, and TPB boundary length is depicted in Figure S7, Supporting Information. It is seen that the electrode samples reconstructed by REMind present low errors generally within ±10% regarding specific area density for three phase pairs, i.e., pore/YSZ, pore/Ni, and Ni/YSZ. The reconstructed tortuosity of the pore phase presents significantly low error (around −1%) compared with the reference samples. For TPB length, most reconstructed samples exhibit an average error −5%. The comprehensive structural analysis proves that REMind can accurately reconstruct electrode samples along the through‐plane direction by reflecting reasonable structural features.

Meanwhile, the cross‐sectional slices along three axes are shown in Figure S8c,d, Supporting Information. The horizontal stripe mainly appears on the sample surface, as shown in the cross‐sectional slices in Figure S8c,d, Supporting Information. These horizontal stripes were not observed in the previous data fusion GAN model^[^ ^30^ ^]^ because the data fusion GAN model employed a 3D generator and a 2D discriminator, allowing more accurate correlation between pixel positions along the thickness direction. However, REMind only employs a 2D generator to generate a 3D sample slice by slice, which may result in weaker correlation along slices in different positions. Despite the limit in cross‐slice correlation, REMind still shows reasonable accuracy in most areas, as evidenced by the statistical error analyzes along two axes in Figure S8f,g, Supporting Information. Additionally, two possible ways could be employed to improve horizontal stripe: the first is to add padding layers to the convolutional neural layers of the U‐net, as highlighted by Kench et al.^[^ ^17^ ^]^; The second is to train REMind with grayscale images, the common output of FIB‐SEM imaging, allowing smooth reconstruction of the material interface. The discontinuous patterns are improved through a smoothness algorithm, which smooths the interface of three phases by transforming isolated single voxels into their neighbor value. The algorithm is accessible in the section on code availability.

The performance was further compared with a cutting‐edge super‐resolution GAN model developed by Dahari et al.^[^ ^30^ ^]^ who integrated 2D high‐resolution slices and 3D low‐resolution blocks, see Figure S9, Supporting Information. Although REMind and the data fusion GAN model employed different super‐resolution strategies, the generated SOFC electrode microstructures present similar structural characteristics for the nearly same low‐resolution structures as input, as shown in Figure S9, Supporting Information. Due to the different model implementation, the low‐resolution input (32 slices with a size 32^2^ pixels) of REMind does not exactly match the data fusion model (34^3^ voxels), which is possibly the reason leading to differences in the generated microstructures.

However, direct accuracy comparisons between REMind, sliceGAN^[^ ^17^ ^]^ and the data fusion GAN model^30]^ are inappropriate, since these methods operate on fundamentally different inputs and pursue distinct objectives. SliceGAN is designed to maximize microstructural diversity and does not perform reconstruction, whereas Data Fusion jointly leverages a 2D high‐resolution slice and a 3D low‐resolution volume, unlike REMind, which reconstructs a full 3D microstructure from two 2D slices. Moreover, the original data fusion GAN studies did not include a pixel‐wise comparison against true high‐resolution samples; by contrast, the present work explicitly quantifies voxel‐level fidelity between generated and ground‐truth volumes. The presented evaluation provides the first rigorous, pixel‐wise accuracy assessment for this class of reconstruction models.

Furthermore, the comparison regarding the *J*‐*η* curves of the SOFC anode reconstructed by REMind and traditional FIB‐SEM exhibits significant differences, suggesting a more practical way to quantify the reconstructed error by REMind. Notably, the *J*‐*η* curves were obtained using a multi‐scale multi‐physics SOFC electrode model, as detailed in the Experimental Section. It is widely acknowledged that the electrode samples with representative volumes present similar electrochemical performance. However, the electrodes with the same volume of phase fraction can be slightly different in the spatial distribution of materials. In this study, we value the pixel‐wise reconstruction accuracy and use a multi‐scale electrochemical model to reveal the reconstruction difference from a different perspective. Figure 4c displays the *J*‐*η* curves under a range of *L* values. Each curve was averaged across 20 samples with a size 128^2^ × 120 voxels. It is seen from Figure 4c that cases *L* = 4, 6, and 8 have nearly absolute errors 2.5%, −3.3% and −2.7% respectively. While cases *L* = 10 and 12 deviate from the ground truth significantly by an error of 9.5% and 19.9% respectively. A detailed error distribution for cases *L* = 4–12 regarding the operating current density *J* under *η* = 0.12 V is shown in Figure 4e. It demonstrates that the electrochemical performance of the reconstructed samples by REMind is less sensitive to the variation of *L* than pixel‐wise structural analysis, thus larger *L* could be used to accelerate microstructure reconstruction. Notably, the slight error among cases *L* = 4, 6, 8 and ground truth arises because variations in local pixel configurations have minimal impact on the effective properties of the sample, such as structural connectivity, effective conductivity, and active triple‐phase boundaries. Figure 4d further illustrates the ionic potential distribution of two samples: the ground truth and case *L* = 6. The nearly identical ionic potential distributions suggest that the reconstructed sample by REMind can accurately reflect the relevant physics of the microstructures. Here, the predicted electrochemical performance plays a key role in guiding the choice of *L*. According to Figure 4b, the reconstructed SOFC electrode microstructures at a high *L* (e.g., 12) do not show significant differences in structural two‐point correlation coefficients compared to those at a low *L* (e.g., 6); however, as shown in Figure 4e, there is a notable deviation between the two *J*‐ *η* curves, suggesting that a high *L = *12 may lead to reconstructed microstructures with considerably overestimated electrochemical performance than the ground truth.

### 3.3 Demonstration of REMind on Diverse Energy Materials

**Figure 5:**
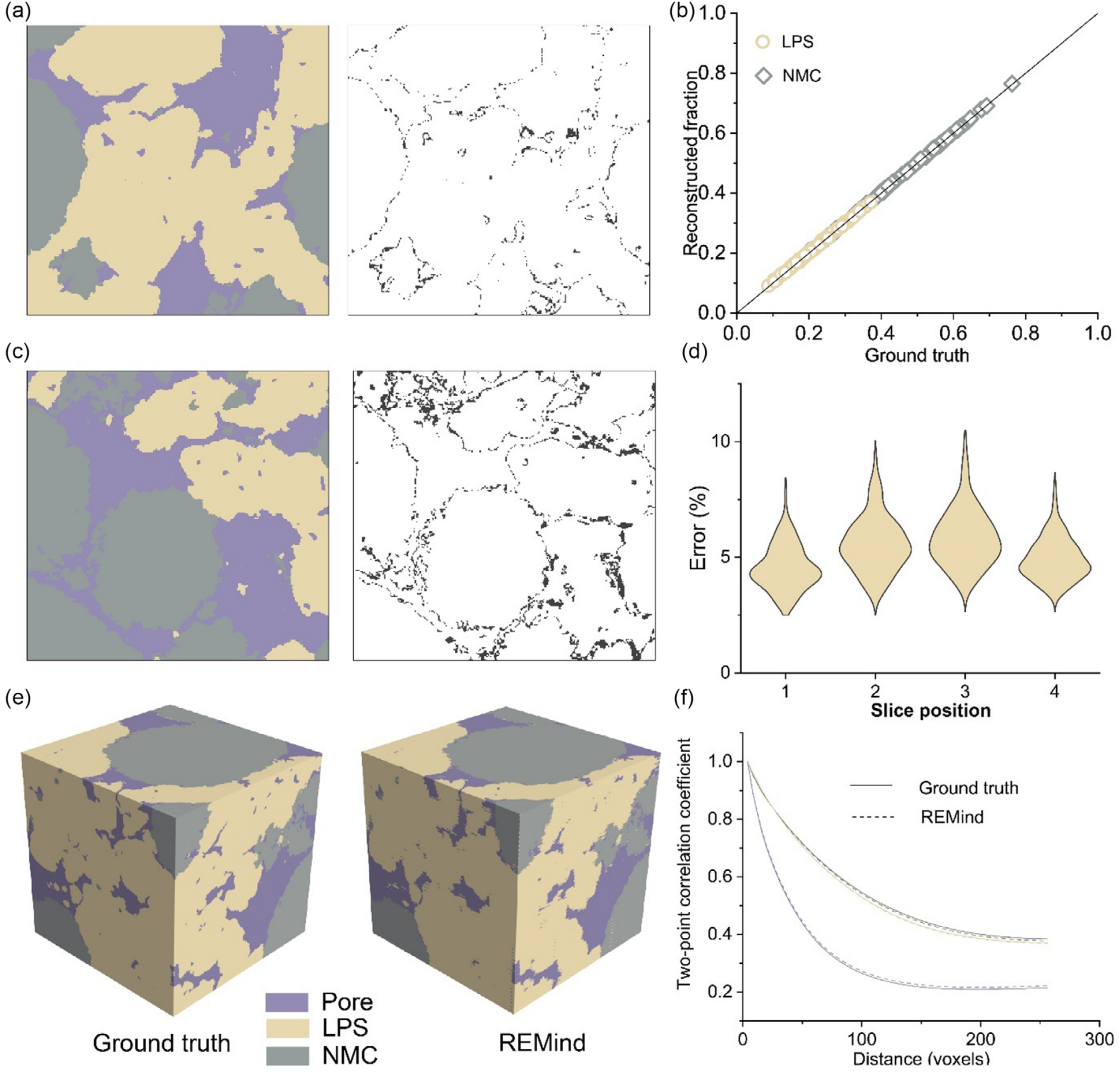
REMind performance of SSB composite electrode reconstruction. a) An in‐plane super‐resolution output (left) and error distribution (right) at stage one. b) The distribution of the volume fractions of 100 blocks from through‐plane reconstruction at stage two. c) The middle slice reconstructed under *L* = 6 (left) and error distribution (right) at stage two. d) The error distribution of 100 reconstructed blocks in each middle slice. e) Two samples (256^3^ voxels) reconstructed by a FIB‐SEM (ground truth) and REMind (*L* = 6). f) Two‐point correlation coefficients of the two samples in (e).

In addition to evaluating REMind on SOFC anode reconstruction, we attempted to explore the potential of REMind for diverse energy materials, particularly for those that provide key sites for electrochemical reactions. We first targeted a composite positive electrode for a thiophosphate SSB.^[^ ^40^ ^]^ The composite electrode consists of polycrystalline NMC622 (LiNi_0.6_Mn_0.2_Co_0.2_O_2_) particles and amorphous Li_3_PS_4_ solid electrolyte (LPS). A FIB‐SEM sample^[^ ^40^ ^]^ was used to train REMind. **Figure** **5**a shows the super‐resolution surface by taking the low‐resolution input from Figure S10a, Supporting Information. The error of the super‐resolution surface is significantly low, further demonstrated by the error distribution of reconstructed volume fraction across 100 surfaces in Figure S10b, Supporting Information. Figure 5,c and S10c–f, Supporting Information, show the through‐plane reconstruction performance under *L* = 6. It is interesting to observe lower reconstruction error both globally and locally (see Figure 5c,d, and 3c–e). This is because the SSB composite positive electrode contains a significant number of large structures, e.g., concentrated NMC particles and LPS electrolytes, and thus the consistency of slices along the through‐plane is better than the SOFC anode. Moreover, the uncertainty of the generation is significantly low except for very few spots, as shown in Figure S10f, Supporting Information. Figure 5e shows a 256^3^‐voxels SSB positive electrode sample reconstructed by REMind. The reconstructed sample is nearly identical to the ground truth regarding its appearance and structural two‐point correlation curve (see Figure 5f). The detailed structures inside the electrode and the reconstruction error distribution along different directions are presented in Figure S11, Supporting Information. Although the current study mainly focuses on the material structure, comparison regarding battery electrochemical performance could be achieved by employing a series of pore‐scale battery models^[^ ^41^, ^42^, ^43^ ^]^ in the future.

**Figure 6:**
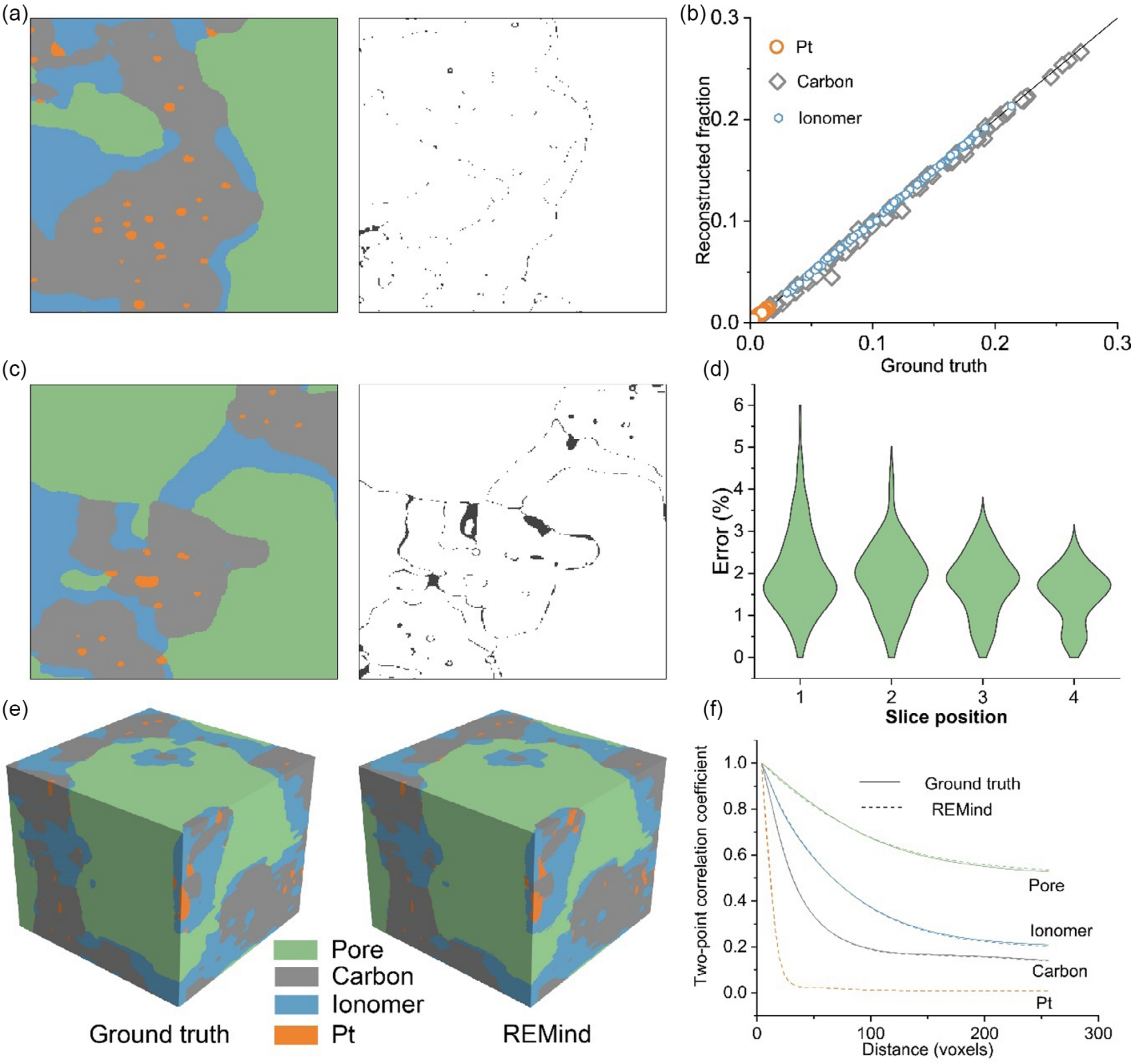
The performance of REMind in reconstructing CLs of proton exchange membrane fuel cells. a) An in‐plane super‐resolution output (left) and error distribution (right) at stage one. b) The distribution of the volume fractions of 100 blocks from through‐plane reconstruction at stage two. c) The middle slice reconstructed under *L* = 6 (left) and error distribution (right) at stage two. d) The error distribution of 100 reconstructed blocks in each middle slice. e) Two samples (256^2^ × 234 voxels) reconstructed by a FIB‐SEM (ground truth) and REMind (*L* = 6). f) Two‐point correlation coefficients of the two samples in (e).

We further demonstrated the performance of REMind on catalyst layers (CL) of proton exchange membrane fuel cells,^[^ ^42^ ^]^ which host complex heat and mass transfer and electrochemical reactions. **Figure** **6**a demonstrates the accuracy of an in‐plane super‐resolution surface by taking a low‐resolution input in Figure S12a, Supporting Information. The error distribution of reconstructed phase fraction across 100 surfaces further verifies the in‐plane super‐resolution accuracy. The through‐plane reconstruction is illustrated in Figure 6c,d, and S12c–f, Supporting Information. Although the overall error is low (less than 10%), some accumulated errors arise in the pore or carbon regions in the middle slices (Figure 6c). This is possibly due to the sharp variation of materials around the error, as highlighted in Figure S12c, Supporting Information. The reconstructed error along the through‐plane direction is lower than that of SSB. This is because the phase fraction of pore in CLs is significantly high (averaged 67%) and thus is more tolerant of the errors of the pore phase. The generated uncertainty is as low as the SSBs, as shown in Figure S12f, Supporting Information. The ultimately reconstructed CL sample proves nearly identical to the ground truth, as shown in Figure 6e,f, further demonstrating the performance of REMind on diverse energy materials.

It is worth noting that the current version of REMind focuses on using the diffusion model to generate electrode microstructures. Recent breakthroughs in transfer learning‐based approaches provide a practical avenue to generate electrode microstructures with arbitrary geometries.^[^ ^44^, ^45^, ^46^, ^47^ ^]^ For instance, Xu et al. pioneered a unique generative AI model by developing a novel transfer learning‐based framework tailored for microstructure reconstruction within arbitrary complex geometries. They creatively repurposed VGG19, a pre‐trained 2D convolutional neural network to iteratively generate 3D volumes statistically equivalent to a single 2D microstructural reference image.^[^ ^44^ ^]^ They further proposed a generative graph neural network to design connectivity‐guaranteed porous metamaterials.^[^ ^45^ ^]^ Compared with the transfer learning‐based approaches, REMind is limited to generative electrodes with arbitrary geometries, e.g., irregular electrode geometries in circular and tubular SOFCs, which are important in real engineering applications. However, REMind could be improved to generate with arbitrary geometries by integrating geometry‐aware conditioning mechanisms into its diffusion backbone, such as voxelised masks of the target domains, or adopting modular generation strategies like patch‐wise synthesis combined with seamless transition techniques.

It is true that both sliceGAN^[^ ^17^ ^]^ and the data fusion GAN model^[^ ^30^ ^]^ employ fully convolutional layers and thus can accept inputs of varying spatial dimensions once trained. However, because they rely on a 3D generator (with an accompanying 2D discriminator), their time and memory complexities scale as *O*(N^3^). In practice, generating larger representative volumes (e.g., moving from 64^3^ to 128^3^ or 256^3^) often requires retraining these models to capture longer‐range correlations, further increasing overhead. In contrast, REMind's reconstruction pipeline scales as *O*(N^2^) in both compute and peak memory, allowing rapid generation of arbitrarily large volumes without requiring massive retraining resources. This quadratic scaling demonstrates clear advantages in scalability and resource efficiency over existing GAN‐based approaches.

The efficiency of REMind during inference is limited compared to GAN‐based generative models,^[^ ^17^, ^30^ ^]^ which can generate hundreds of 3D samples within seconds, even at large output sizes. The limitation arises because REMind requires several minutes for a single sample to denoise a Gaussian field through thousands of steps, with the time linearly increasing as the output size grows. However, REMind benefits from a simpler loss function (e.g., L1 or L2 loss) than the WGAN‐GP loss function, which comprises an adversarial loss plus gradient penalty, presenting an easier way to track the training process.

Notably, REMind employs only 2D convolutional layers throughout its architecture, while SliceGAN^[^ ^17^ ^]^ and the data fusion model^[^ ^30^ ^]^ utilize a 3D generator and a 2D discriminator. As a result, REMind's memory requirements scale with dimensionality as *O* ^2^ rather than *O* ^3^, making it feasible for reconstructing large sample sizes, such as 256^3^ voxels for the nanoporous CLs and composite SSB electrode in this study.

Moreover, REMind is flexible in generating cuboid samples without retraining, e.g., 128^2^ × 256, 128^2^ × 512, etc. These cuboid configurations are valuable for investigating heat, mass, and charge transfer along the electrode thickness direction.

A known limitation of REMind is the appearance of slight discontinuities (i.e., striped artifacts) near the surface of the generated 3D samples. This issue does not arise in SliceGAN or the data fusion model, as both employ 3D generators that capture voxel correlations in three dimensions. As previously discussed in Figure S8, Supporting Information, these stripes can be mitigated using a simple smoothing algorithm.

## 4 Conclusion

3D structure reconstruction via time‐consuming imaging techniques such as FIB‐SEM has significantly contributed to the progress of next‐generation energy materials in various electrochemical energy devices. However, both imaging speed and field of view need to be improved to fulfill new challenges in energy materials, such as high‐throughput characterization. In this work, we introduced a novel generative AI framework, REMind, to overcome the two key limitations of FIB‐SEM. REMind is a two‐stage generative diffusion AI model capable of performing in‐plane surface super‐resolution and through‐plane reconstruction of 3D internal microstructures between two surfaces. The trustworthiness of REMind's output was further enhanced by extensive spatial error analyzes, uncertainty quantification, and multi‐physics electrode modeling. We mainly demonstrated the performance of REMind in reconstructing SOFC electrodes. It was found that the accuracy of REMind depends on the thickness of the block, i.e., the number of slices that are skipped. Meanwhile, the multi‐physics modeling of the reconstructed SOFC electrode indicates that the reconstructed electrode from REMind agrees well with the ground truth, even with fewer milling times regarding its electrochemical performance, i.e., the relationship between operating current density and overpotential. Furthermore, we demonstrated the performance of REMind on diverse energy materials, such as the composite positive electrode of SSB, and CLs of proton exchange membrane fuel cells.

REMind is proven feasible on diverse structures and may potentially act as a key in accelerating the discovery of innovative energy materials. By significantly reducing the number of required FIB‐SEM slices, REMind accelerates 3D microstructure reconstruction while minimizing imaging time and experimental costs. This makes it a highly efficient and cost‐effective alternative to traditional imaging approaches, enabling broader and more frequent microstructural analyzes without excessive resource expenditure. Beyond fuel cells and batteries, REMind's ability to reconstruct complex 3D microstructures could be beneficial in a wide range of applications, including analyzing the structure of solar cells and biomaterials characterization, etc. Its capability to generate high‐fidelity 3D structures from limited experimental data paves the way for accelerated materials discovery and device optimization across multiple scientific and engineering disciplines.

## 5 Experimental Section

5.1

5.1.1

##### Generative Diffusion Model

We employed a denoising diffusion probabilistic model (DDPM) model^[^ ^48^ ^]^ in the study. A DDPM model is a powerful probabilistic generative AI model that has demonstrated success in data generation and image super‐resolution.^[^ ^49^, ^50^ ^]^ A typical DDPM model usually consists of two processes, i.e., forward and backward diffusion processes. In the forward process, the original data sample *x*_0_ is converted to random Gaussian noise through a Markov chain, represented as follows

(1) $$p_{\theta }\left(x_{0:T}\right):=p\left(x_{T}\right)\prod_{t=1}^{T}p_{\theta }\left(x_{t-1}\text{|}x_{t}\right)$$

where *p*(*x*_T_) follows a standard Gaussian distribution. The transition probability $p_{\theta }\left(x_{t-1}\text{|}x_{t}\right)$ is chosen as:

(2) $$p_{\theta }(x_{t-1}\text{|}x_{t},c):=N(x_{t-1};\mu_{\theta }(x_{t},t),\sum_{\theta }(x_{t},t,c))$$

where *c* is the conditions during the backward diffusion process. Notably, REMind is a classifier‐free conditional diffusion model. Rather than relying on a separate classifier to guide the reverse diffusion process, REMind directly incorporates the conditioning information (low‐resolution bottom and top surfaces, alongside the slice position along the thickness direction) into the denoising network. Specifically, the position of each slice in a given block is directly embedded into the network by using a sinusoidal function, ensuring that REMind can distinguish different slices between the top and bottom slides. Additionally, the top and bottom slices of a given block are concatenated with time step and slice position embeddings into the network, further serving as conditional signals. More fundamental understandings of conditional diffusion models can be accessed in recent studies.^[^ ^51^, ^52^ ^]^ Meanwhile, the technical details of conditional diffusion implementation are available in the public code. A DDPM model can use a neural network to estimate the inverse of the forward process, i.e., backward diffusion process, and thus the model is capable of generating original data from noise. Therefore, a forward process of *T* time steps can be further constructed as

(3) $$q\left(x_{1:T}\text{|}x_{0}\right):=\prod_{t=1}^{T}q\left(x_{t}\text{|}x_{t-1}\right)$$

where $q\left(x_{t}\text{|}x_{t-1}\right)$ is modelled as a Gaussian noise distribution $N\left(x_{t};\sqrt{1-\beta_{t}}x_{t-1},\beta_{t}I\right)$. Here, $\beta_{t}$ is a hyperparameter of the neural network in the model. The choice of $\sqrt{1-\beta_{t}}$ can scale the variance of noise added to each step of the forward diffusion process to ensure that the original data is progressively corrupted while maintaining some structures in the earlier steps.

In the backward diffusion process, a neural network is trained to predict the noise added to the original data sample *x*_0_ by minimizing the following simplified training objective

(4) $$L_{t}^{\text{simple}}=E_{\text{t∼}[1,T],x_{0},\varepsilon }[∥\varepsilon_{t}-\varepsilon_{\theta }(\sqrt{{\bar{\alpha }}_{t}}x_{0}+\sqrt{1-{\bar{\alpha }}_{t}}\varepsilon_{t},t,c){∥}^{2}]$$

where ${\bar{\alpha }}_{t}$ is $1-\beta_{t}$, ${\bar{\alpha }}_{t}$ is a cumulative product $\prod_{t=1}^{T}\alpha_{i}$, representing the fraction of information retained from the original data after t steps. $\varepsilon_{t}\approx N\left(0\right)$ is the actual Gaussian noise added at time step t during the forward process. $\varepsilon_{t}$ is a known term, as the forward diffusion process is explicitly defined. $\varepsilon_{\theta }\left(\cdot \right)$ is the noise predicted by the neural network parametrized by θ, given the input $\sqrt{{\bar{\alpha }}_{t}}x_{0}+\sqrt{1-{\bar{\alpha }}_{t}}\varepsilon_{t}$ at time step t. The ultimate loss function measures the squared error between the actual noise $\varepsilon_{t}$ and the noise predicted by the neural network $\varepsilon_{\theta }$. Minimizing the loss function ensures the model learns to predict the added noise accurately, given the condition *c*. Notably, *c* only represents low‐resolution images in the in‐plane super‐resolution stage. However, *c* consists of three types of information in the through‐plane super‐resolution stage, i.e., slice position, top, and bottom super‐resolution images of a given block.

##### Model Architecture and Training

A U‐net was adopted in the DDPM to predict the added noise in the forward diffusion process. The architecture and model parameters of a 2D U‐net neural network are depicted in Figure S13, Supporting Information. Particularly, a self‐attention layer is added to the bottleneck of the U‐net to improve the model accuracy. At the in‐plane super‐resolution stage, a low‐resolution conditional image was rescaled to the size of the super‐resolution image. At the through‐plane super‐resolution stage, the slice position *i* in a given block [L_1_, L_2_ …L_i_…L_N_] is encoded into the model input by using a sinusoidal positional encoding. The embedding of slice position allows for training a 2D DDPM instead of a 3D one to generate internal structures sequentially in blocks and thus make the model significantly efficient.

The DDPM model was developed in PyTorch. A Mean Absolute Error loss function was employed to measure the error between the predicted and added noise. The Adam optimizer with a learning rate 5 × 10^−5^ was used to update the model parameters. The batch size was 64 for all cases in the study. The input image size varied for cases, depending on the size of the region of interest. For instance, 128^2^ and 256^2^ pixels were determined for the SOFC anode and solid‐state Li‐ion electrode. For the preparation of data, 20 000 and 200 pairs of low‐ and high‐resolution 2D images were obtained for the training and testing dataset, respectively, by randomly extracting slices from a FIB‐SEM database of SOFC,^[^ ^53^ ^]^ cryo‐genetic Electron Microscopy (EM) database of CLs of proton exchange membrane fuel cell (PEMFC),^[^ ^54^ ^]^ and FIB‐SEM database of SSB.^[^ ^40^ ^]^ Likewise, 20 000 and 200 blocks with a given slice number were prepared using the same way for the training and testing dataset of the through‐plane training. The convergence study was conducted across five training datasets, including 1,000, 5,000, 10 000, 15 000, and 20 000 samples, respectively. The reconstruction errors of REMind on different datasets over 200 test samples are 13.99%, 13.39%, 13.32% and 13.1% under *L* = 6, respectively. Although REMind performs well in small datasets, the dataset size 20 000, chosen as a large training dataset, typically enhances the ability of the model to be more robust and reliable, reducing the risk of overfitting to small datasets. Notably, there is no data leakage during the model training because only the top and bottom surfaces are conditioned with the input Gaussian noise during the forward process of the neural network. The model was trained on an A100 NVIDIA graphics processing unit until the decrease rate of the loss value is less than 10% every 1000 steps. For in‐plane super‐resolution and through‐plane data reconstruction, the model was trained at 3,000 epochs. The reserved graphics processing unit (GPU) memory for stages 1 and 2 of REMind is 20.06 and 21 G, respectively, regardless of the size of the super‐resolution factor. Meanwhile, the computational time for reconstructing a single slice (128^2^ pixels) is 24 s, which is independent of the size of *L* and the super‐resolution factor. This is because the model architecture and model parameters remain constant, as depicted in Figure S13, Supporting Information. The overall reconstruction accuracies are 8.0% (*L* = 4), 13.1% (*L* = 6), 19.4% (*L* = 8), 25.9% (*L* = 10), 31.6% (*L* = 12). The super‐resolution accuracies under the factor 8× and 16× are around 25% and 45%, which significantly undermine the through‐plane reconstruction accuracy at stage 2. Thus, this study only focuses on the super‐resolution factor 4×.

##### Evaluation Metrics

Uncertainty quantification of the output of the through‐plane data reconstruction is achieved by using entropy, which is defined by a discrete probability given by
(5)

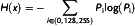

where *P*_i_ is the probability of each pixel value in the generated samples (200 samples in the study) in each pixel location. For the SOFC anode, the pixel values include 0, 128, and 255, which refer to pore, nickel, and YSZ, respectively. Higher entropy indicates high uncertainty, while lower entropy indicates more certainty. The calculation of structural properties, including specific area density, TPB, and tortuosity, was implemented by using TauFactor.^[^ ^39^ ^]^

Apart from quantifying the uncertainty of generated samples, we employed a previously developed multi‐scale, multi‐physics SOFC electrode model^[^ ^55^ ^]^ to evaluate the errors of electrochemical performance, i.e., *J*‐*η* curves, which reveal the variation of operating current density *J* (A cm^−2^) against overpotential *η* (V). The conduction of ions and electrons in the YSZ and Ni phases, respectively, is modeled by the charge conservation equations presented in Equation (6) and (7).

(6) $$\tau_{\text{con}}\frac{\partial \varphi_{\text{ele}}}{\partial t}=\nabla \cdot \left(k_{\text{ele}}\nabla \varphi_{\text{ele}}\right)-\dot{J}$$

(7) $$\tau_{\text{con}}\frac{\partial \varphi_{\text{ion}}}{\partial t}=\nabla \cdot \left(k_{\text{ion}}\nabla \varphi_{\text{ion}}\right)+\dot{J}$$

where $\tau_{\text{con}}$ is a defined capacitance per unit volume, which is used to relax the change of ionic potential $\varphi_{\text{ion}}$ (V) and electronic potential $\varphi_{\text{ele}}$(V) resulting from the large electrochemical reaction rate $\dot{J}$ (A m^−3^). $k_{\text{ion}}$(S m^−1^) and $k_{\text{ele}}$(S m^−1^) are ionic and electronic conductivities of the YSZ and Ni phases, respectively. The values for electronic/ionic conductivity can be found in Ref. [55]. The electrochemical reaction occurring at the TPB sites is described by the Butler‐Volmer (BV) equation as shown below:

(8) $$\dot{J}=j_{0}\left[\exp\left(\frac{\alpha nF\eta }{\text{RT}}\right)-\exp\left(-\frac{\beta nF\eta }{\text{RT}}\right)\right]$$

where *T* (K) is the operating temperature, *F* (C mol^−1^) is the Faraday constant, and *R* (J K^−1^ mol^−1^) is the universal gas constant. *η* (V) represents the overpotential, which is the sum of the activation overpotential and the concentration overpotential. The transfer coefficients are set as *α* = 1 and *β* = 0.5.^[^ ^56^ ^]^ This BV equation was also employed by Li et al.^[^ ^56^ ^]^ in a 3D pore‐scale SOFC anode model. The exchange current density $j_{0}$(A m^−2^) is fitted from the patterned anode experiments of Boer,^[^ ^57^ ^]^ as follows:

(9) $$j_{0}=31.4p_{H_{2}}^{-0.03}p_{H_{2}O}^{0.4}\exp\left(-\frac{E_{\text{act}}}{\text{RT}}\right)$$

where $p_{H_{2}}$ (Pa) and $p_{H_{2}O}$ (Pa) are the partial pressures of hydrogen and water vapor, respectively. The value of the activation energy *E*_act_ (J mol^−1^) in Equation (9) is 1.52 × 10^5^. The Knudsen and molecular diffusion are considered in the current pore‐scale model, and the species equation is described as follows:

(10) $$\frac{\partial \left(\rho Y_{i}\right)}{\partial t}+\nabla \cdot \left(-\rho D_{i}\nabla Y_{i}\right)=\{\begin{array}{c} 0\text{ pore}\\ S_{i}^{\text{tpb}}\text{ TPB sites} \end{array}$$

where $Y_{i}$ is the local mass fraction of each species (H_2_ and vapor in the study), *ρ* (kg m^−3^) the density, and $D_{i}$ (m^2^ s^−1^) the species diffusivity. The mass source term $S_{i}^{\text{tpb}}$(kg m^−3^ s^−1^) is related to the electrochemical reactions in the anode layer and is only active at the TPB sites. The value of $S_{i}^{\text{tpb}}$ is calculated as follows

(11) $$S_{H_{2}}^{\text{tpb}}=-\frac{\dot{J}}{2F}M_{H_{2}}$$

(12) $$S_{H_{2}O}^{\text{tpb}}=\frac{\dot{J}}{2F}M_{H_{2}O}$$

where $M_{H_{2}}$ (kg mol^−1^) and $M_{H_{2}O}$ (kg mol^−1^) are molar mass of H_2_ and H_2_O, respectively. The pore‐scale model is implemented in the open‐source computational fluid dynamics platform OpenFOAM. The operating temperature and inlet gas conditions for the SOFC anode in this study were chosen as 1,073 K, 3% H_2_O, and 97%H_2_, respectively.

## Supporting Information

Supporting Information is available from the Wiley Online Library or from the author.

## Conflict of Interest

The authors declare no conflict of interest.

## Author Contributions

**Zhiqiang Niu**: conceptualization (equal); data curation (equal); formal analysis (equal); funding acquisition (equal); investigation (equal); methodology (equal); resources (equal); software (equal); validation (lead); visualization (lead); writing—original draft (lead). **Zhaoxia Zhou**: data curation (supporting); methodology (supporting); writing—review & editing (supporting). **Patrice Perrenot**: data curation (equal); writing—review & editing (supporting). **Claire Villevieille**: data curation (equal); writing—review & editing (supporting). **Wanhui Zhao**: funding acquisition (equal); methodology (supporting); writing—review & editing (supporting). **Qiong Cai**: conceptualization (equal); data curation (equal); investigation (equal); methodology (equal); resources (equal); supervision (equal); writing—review & editing (equal). **Valerie J. Pinfield**: formal analysis (equal); investigation (equal); methodology (equal); resources (equal); supervision (equal); writing—review & editing (equal). **Yun Wang**: conceptualization (equal); formal analysis (equal); investigation (equal); methodology (equal); resources (equal); supervision (equal); writing—review & editing (equal).

## Supporting information

Supplementary Material

## Data Availability

The data that support the findings of this study are available from the corresponding author upon reasonable request.
