# Supplementary material for: Seeing the Middle: Reconstructing 3D Internal Electrode Microstructures from Low‐Resolution Surfaces with Generative Diffusion Artificial Intelligence
Source: Small Sci. 2025 Sep 23;5(11):2500414. doi: 10.1002/smsc.202500414 (PMC12622559; doi:10.1002/smsc.202500414)
Supplement: Supplementary file 1 — Supplementary Material [file SMSC-5-2500414-s001.pdf]

Supporting Information

Seeing the Middle: Reconstructing 3D Internal Electrode Microstructures from Low-resolution Surfaces with Generative Diffusion AI

*Zhiqiang Niu**, *Zhaoxia Zhou, Patrice Perrenot, Claire Villevieille, Wanhui Zhao, Qiong Cai**, *Valerie J. Pinfield, Yun Wang**

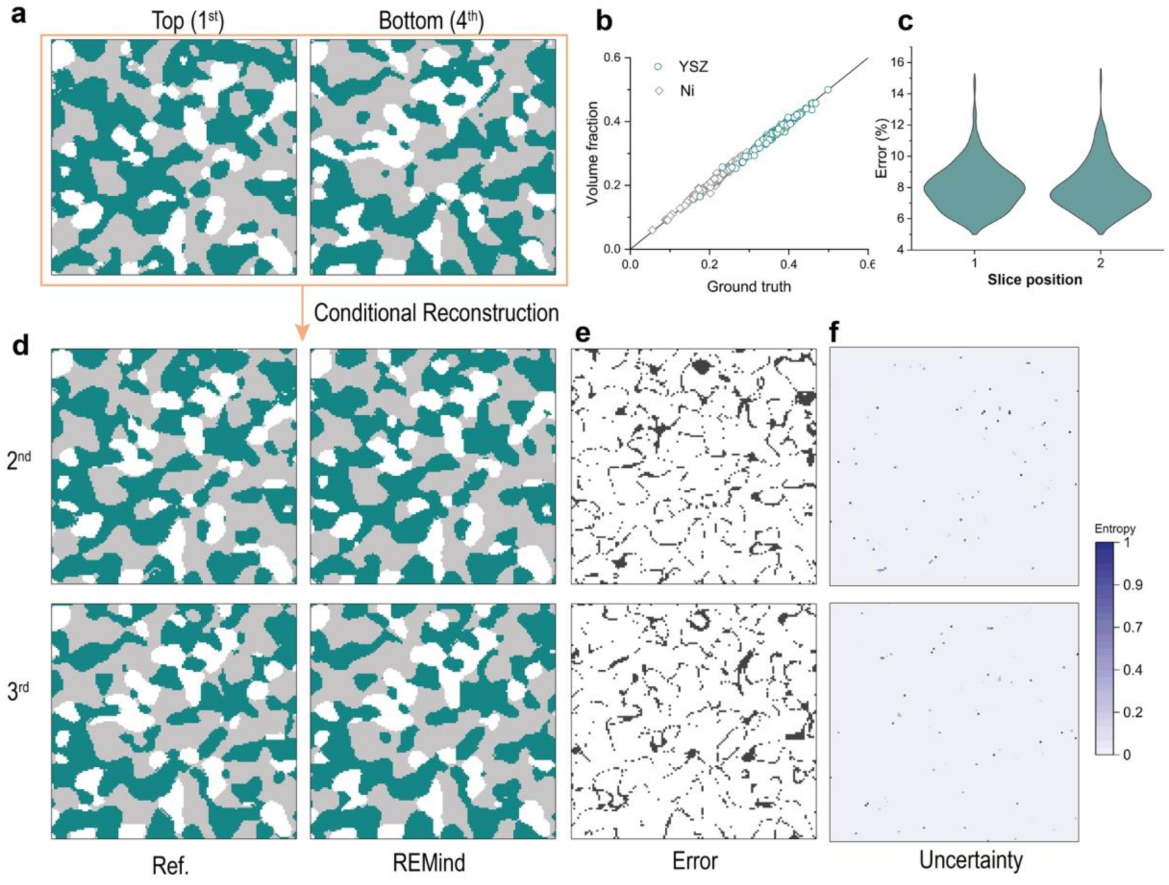

Figure S1 The performance of REMind under L=4. (a) Top and bottom slices of a given chunk provided as conditional input. (b) Distribution of the generated volume fraction of pore and YSZ across 200 samples. (c) Spatial averaged error distributions along the through-plane direction. (d) Ground truth and reconstructed internal microstructures of the given chunk conditioned on the surface information in (a). (e) Spatial error distributions along the through-plane. (f) Entropy map for uncertainty quantification.

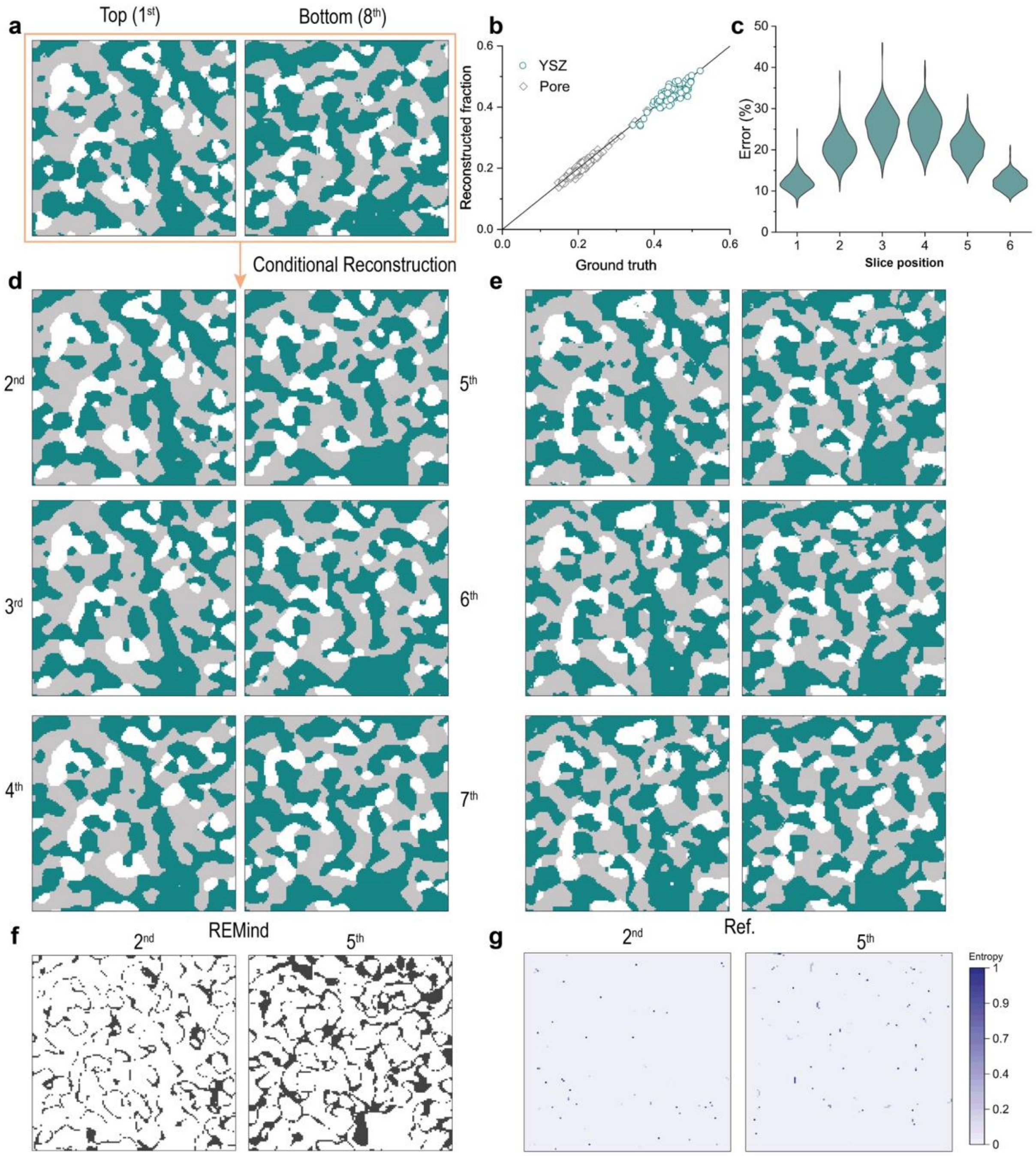

Figure S2 The performance of REMind under L=8. (a) Top and bottom slices of a given chunk provided as conditional input. (b) Distribution of the generated volume fraction of pore and YSZ across 200 samples. (c) Spatial averaged error distributions along the through-plane direction. (d) Ground truth and reconstructed internal microstructures of the given chunk conditioned on the surface information in (a). (e) Spatial error distributions along the through-plane. (f) Entropy map for uncertainty quantification.

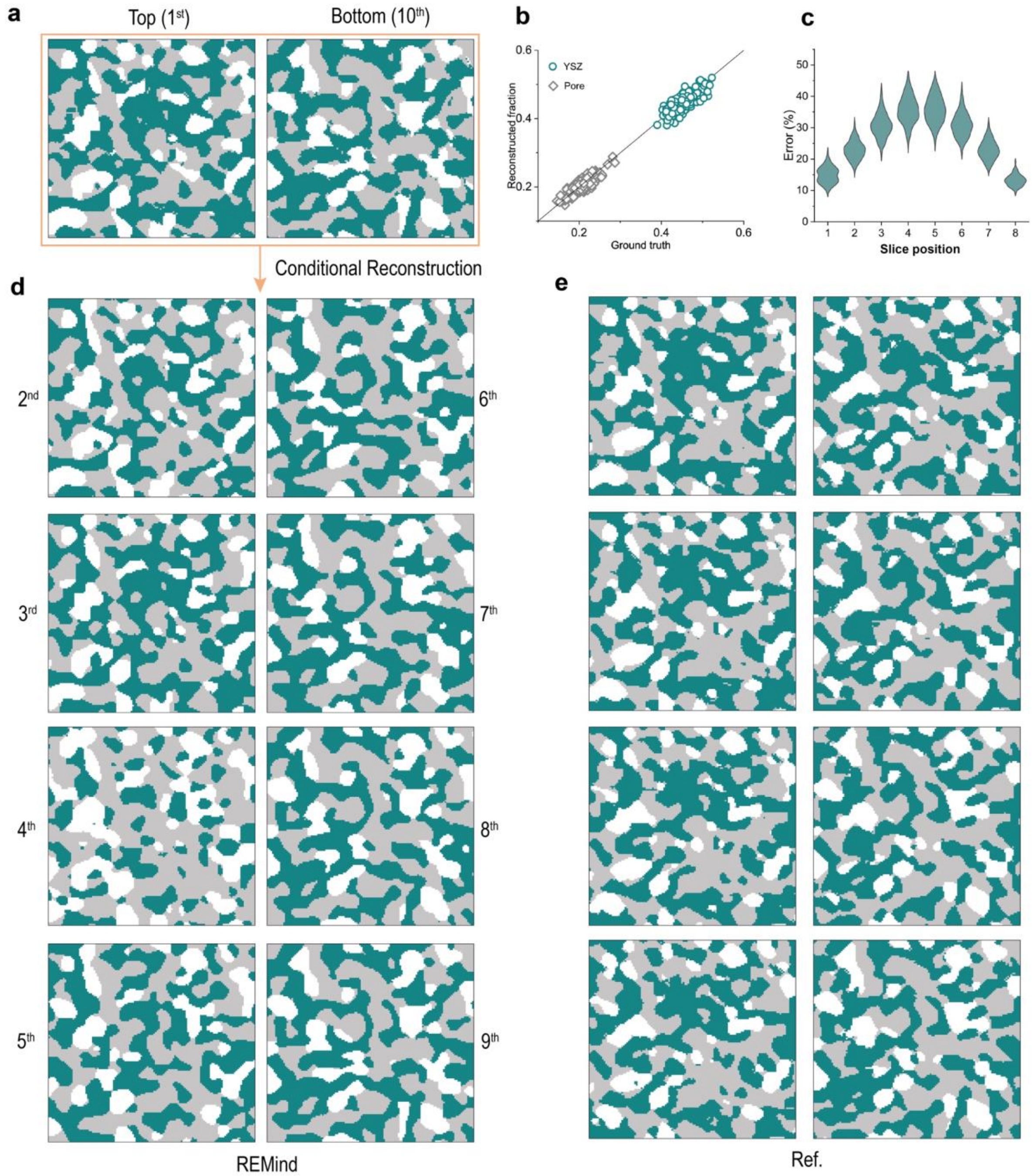

Figure S3 The performance of REMind under L=10. (a) Top and bottom slices of a given chunk provided as conditional input. (b) Distribution of the generated volume fraction of pore and YSZ across 200 samples. (c) Spatial averaged error distributions along the through-plane direction. (d) Ground truth and (e) reconstructed internal microstructures of the given chunk conditioned on the surface information in (a).

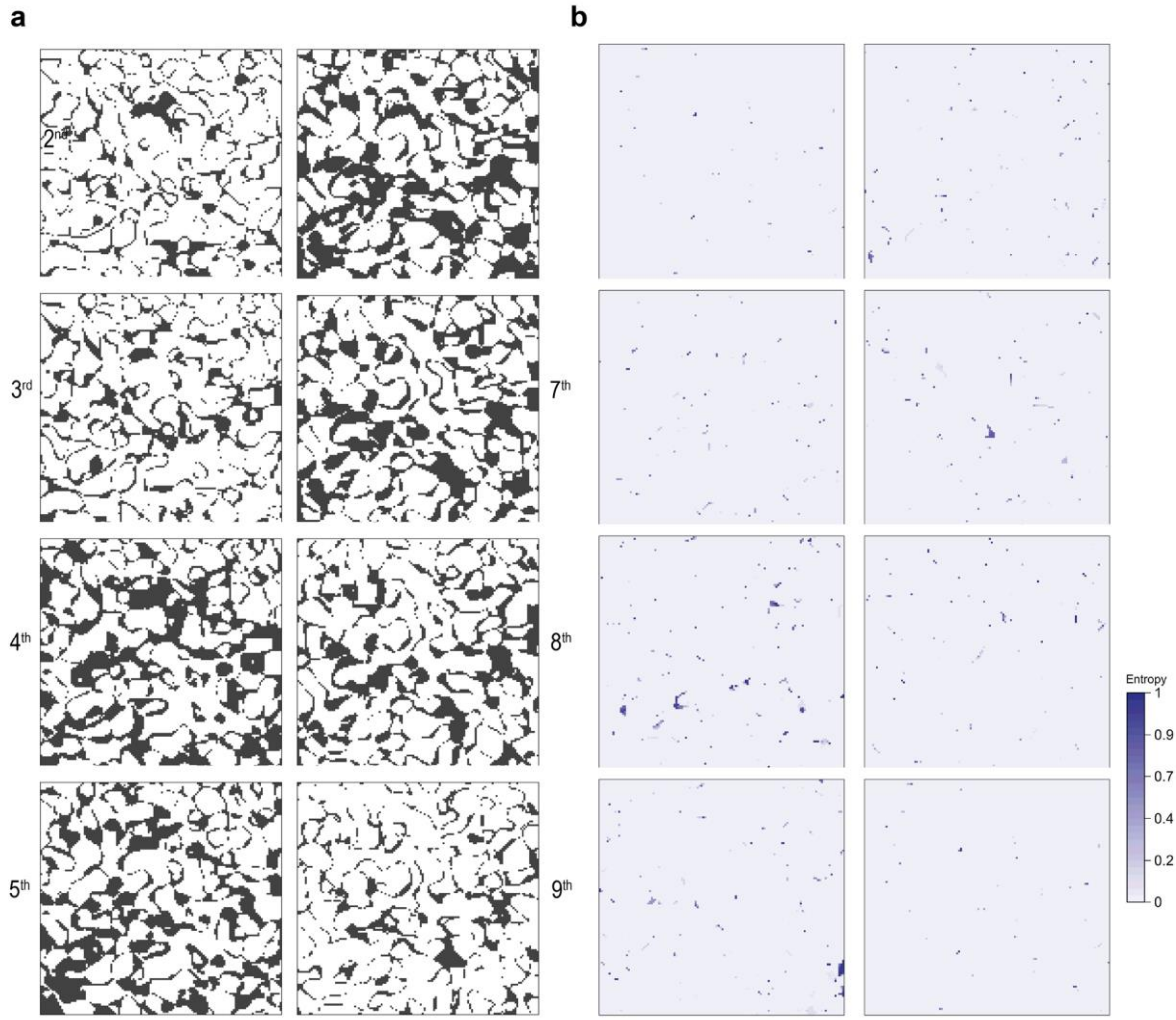

Figure S4 The performance of REMind under *L*=10. (a) Spatial error distributions along the through-plane. (b) Entropy map for uncertainty quantification.

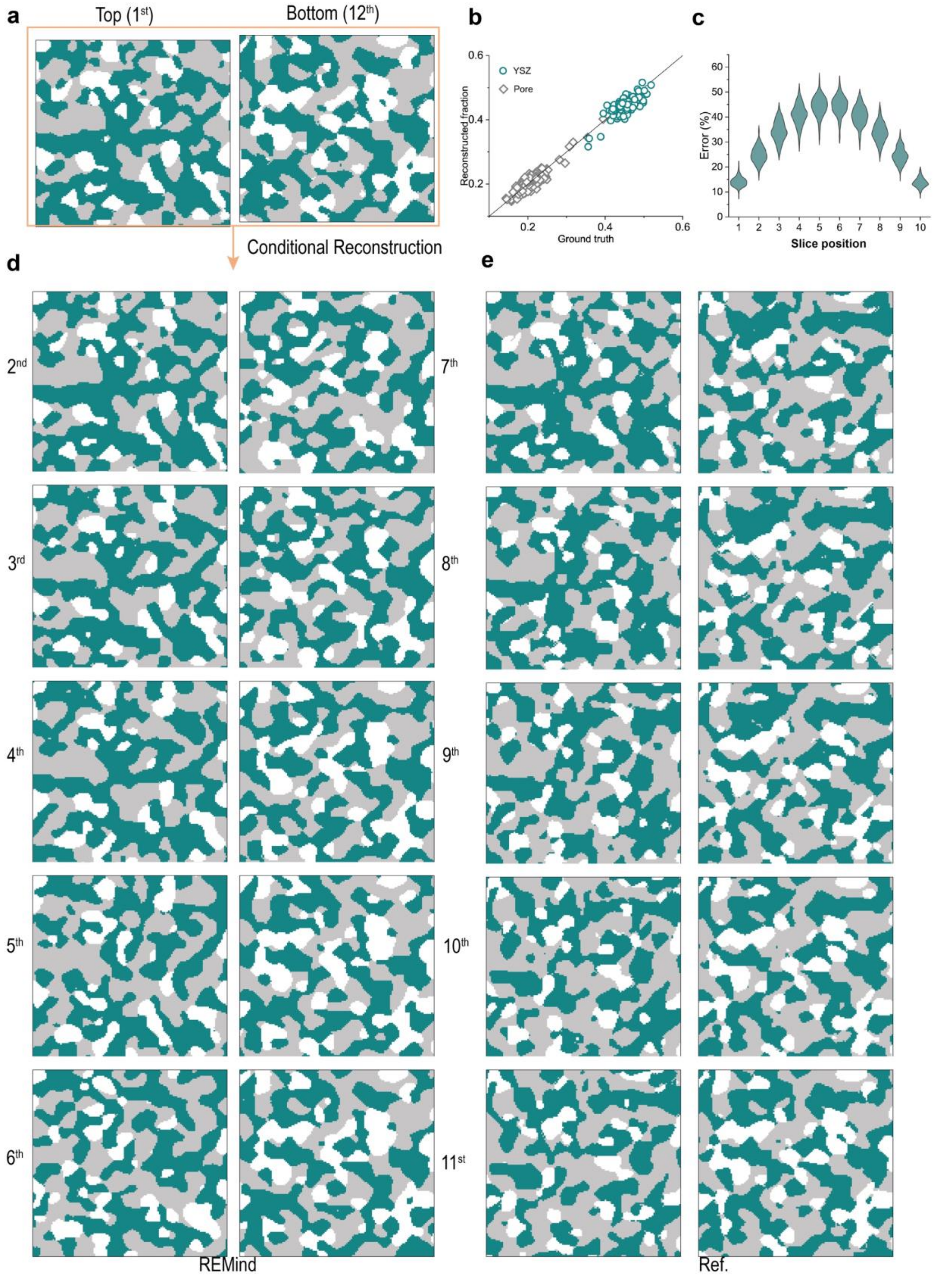

Figure S5 The performance of REMind under L=12. (a) Top and bottom slices of a given chunk provided as conditional input. (b) Distribution of the generated volume fraction of pore and YSZ across 200 samples. (c) Spatial averaged error distributions along the through-plane direction. (d) Ground truth and (e) reconstructed internal microstructures of the given chunk conditioned on the surface information in (a).

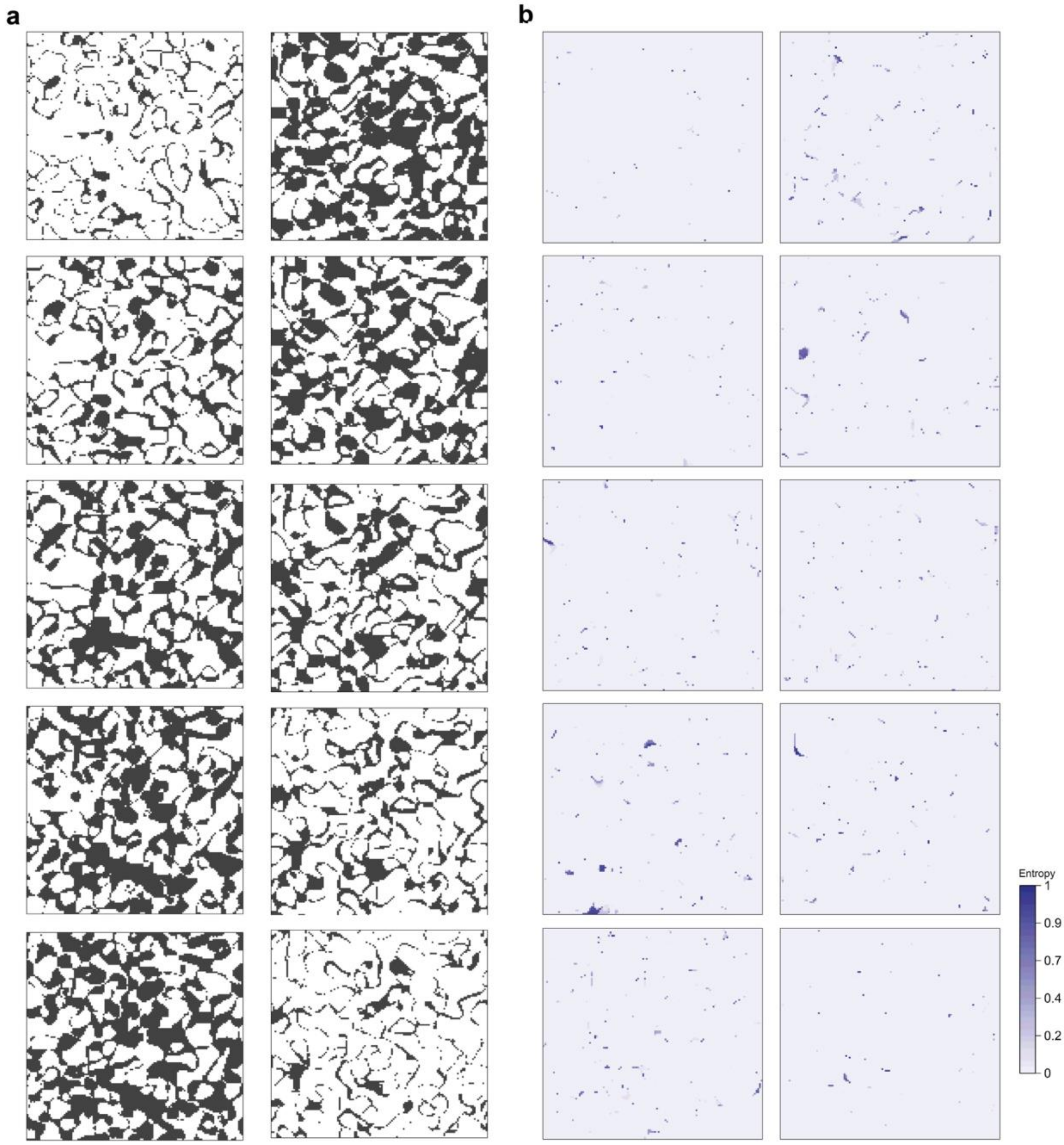

Figure S6 The performance of REMind under L=12. (a) Spatial error distributions along the through-plane. (b) Entropy map for uncertainty quantification.

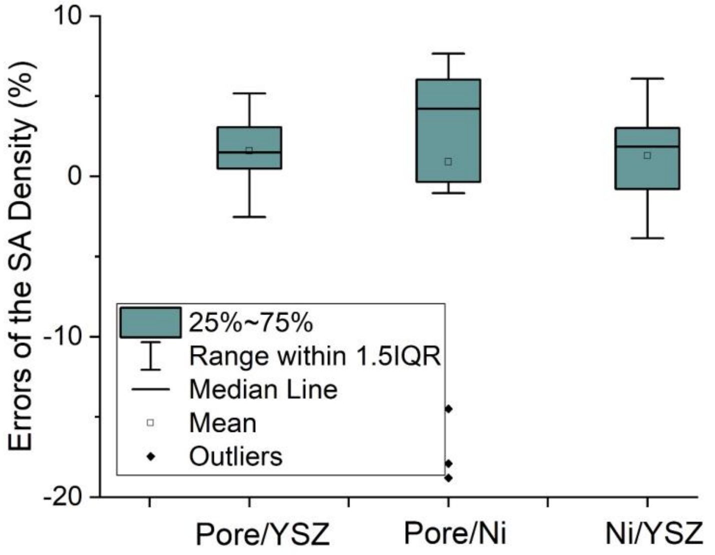

10
0
-10
-20
Errors of the SA Density (%)
25%~75%
Range within 1.5IQR
Median Line
Mean
Outliers
Pore/YSZ
Pore/Ni
Ni/YSZ

(a)

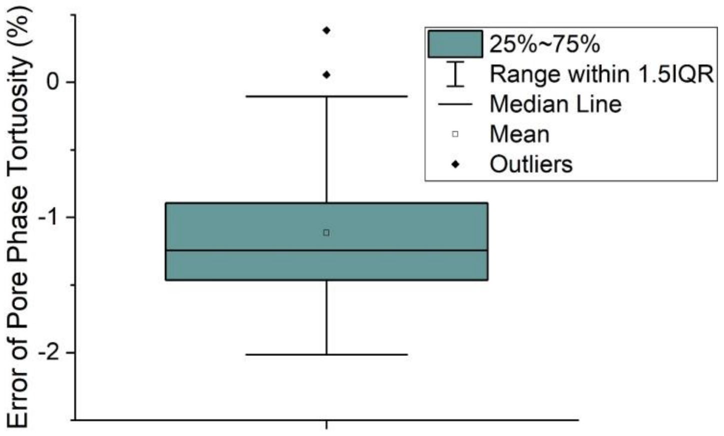

0
-1
-2
Error of Pore Phase Tortuosity (%)
25%~75%
Range within 1.5IQR
Median Line
Mean
Outliers

(b)

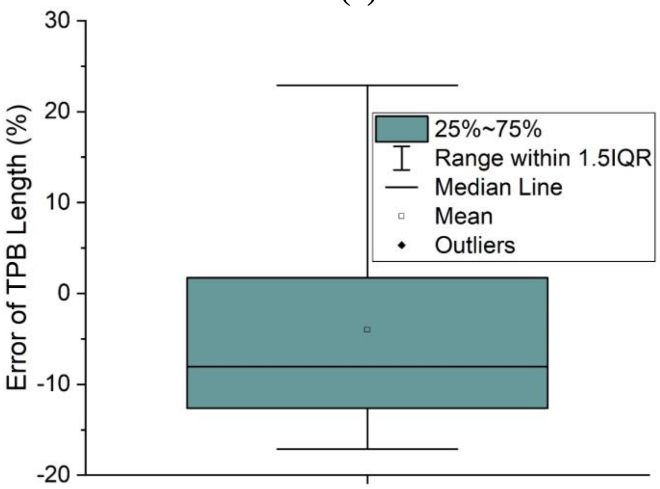

30
20
10
0
-10
-20
Error of TPB Length (%)
25%~75%
Range within 1.5IQR
Median Line
Mean
Outliers

(c)

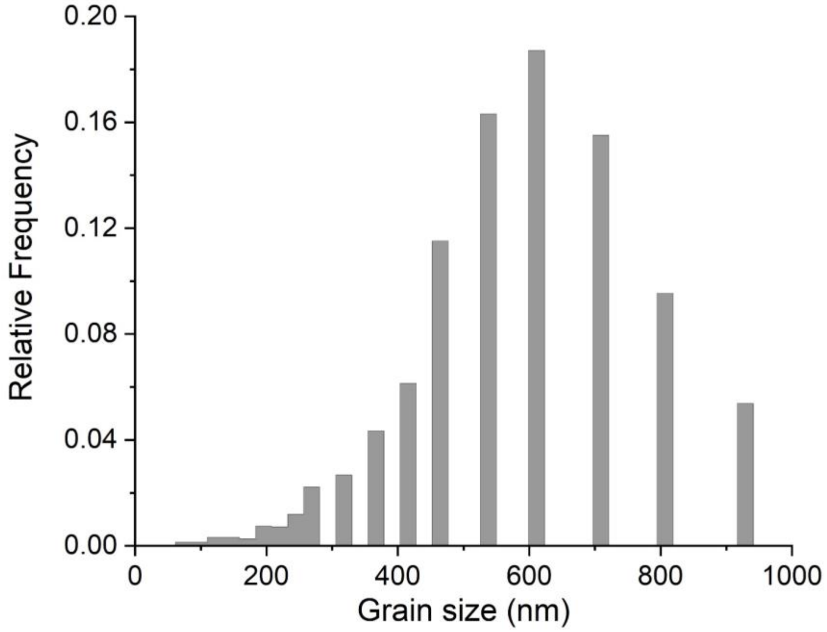

(d)

Figure S7 The error statistics of between the reference and reconstructed electrode microstructures (*L*=6) regarding a series of structural properties (a) Specific area (SA) density, (b) The tortuosity of the pore phase, and (c) the TPB length. 20 samples with the size $128^2 \times 120$ voxels were analyzed in total. (d) The grain size distribution of the real 3D SOFC electrode sample reconstructed by FIB-SEM.

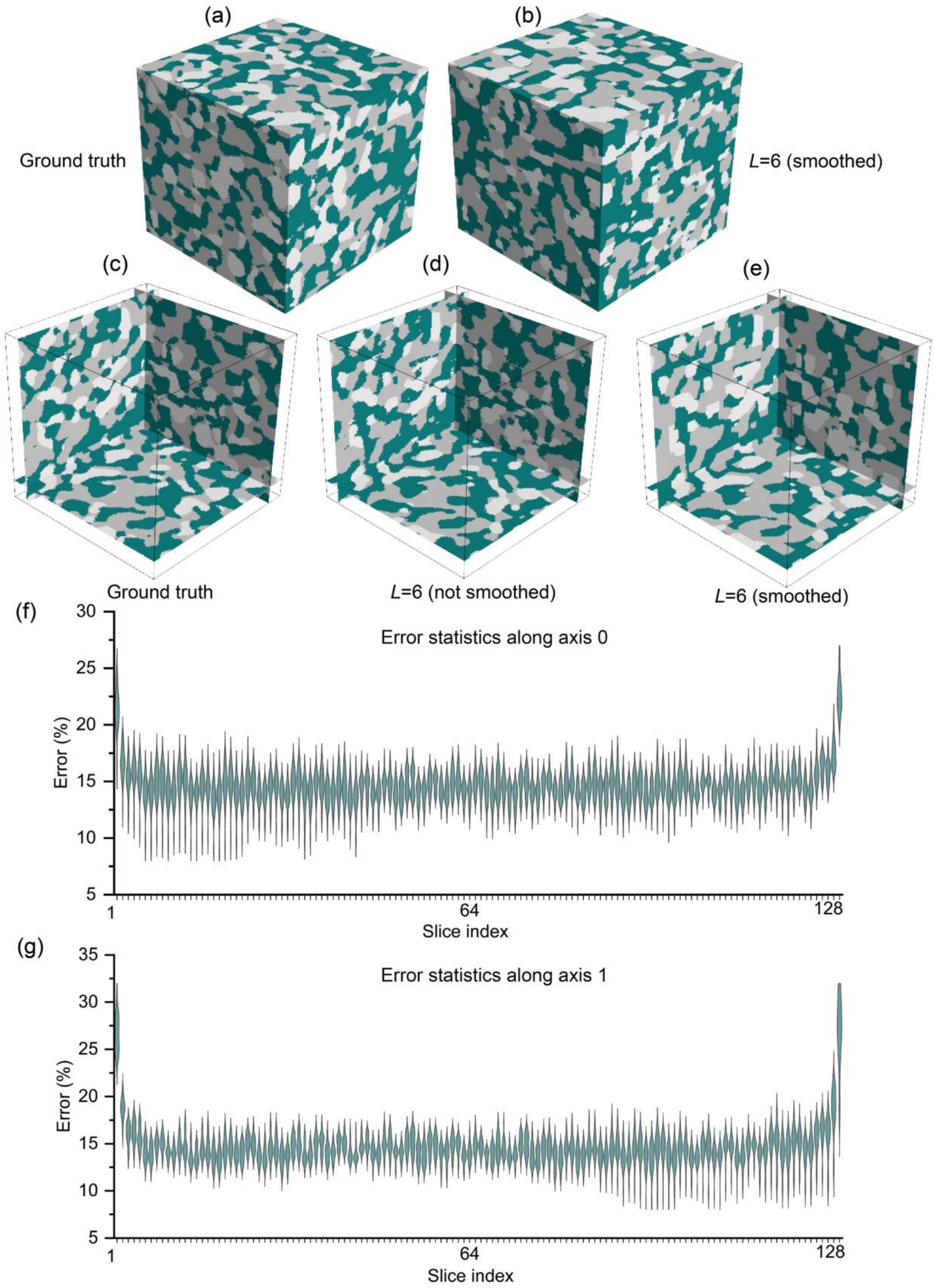

Figure S8 Cross-sectional error analysis and the performance of the smoothness algorithm of discontinuous patterns in the reconstructed SOFC electrode samples. (a) The ground truth for the comparison; (b) The reconstructed sample, refined using the smoothness algorithm; (c-e) Cross-sectional slices of the ground truth, the reconstructed sample without the smoothness, and the reconstructed sample refined by the smoothness algorithm; (f) and (g) Error statistics

across 20 reconstructed samples across axis 1 and axis 2 (the in-plane direction is denoted as axis 0).

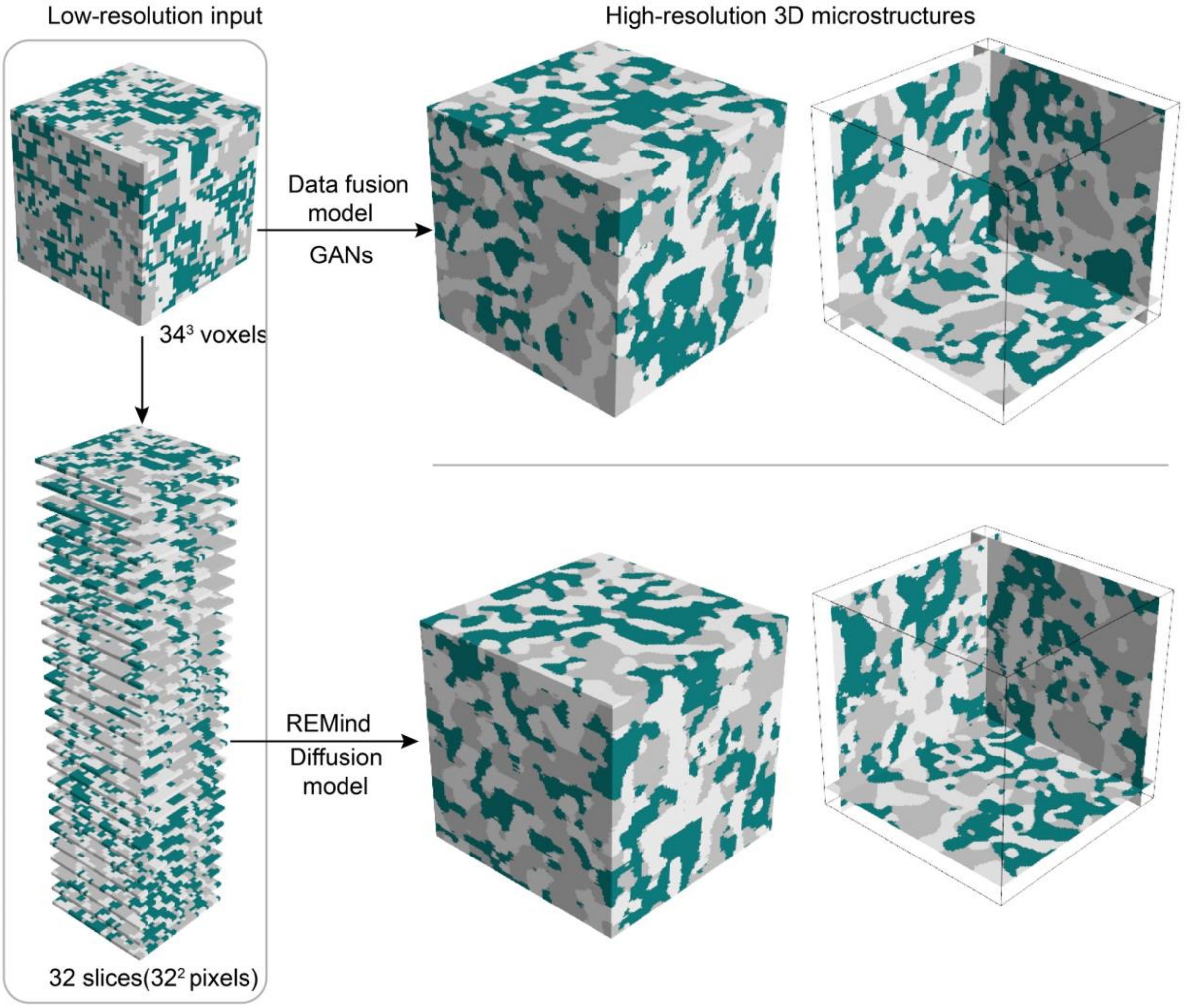

Figure S9 Comparison of REMind and the data fusion GAN model [18].

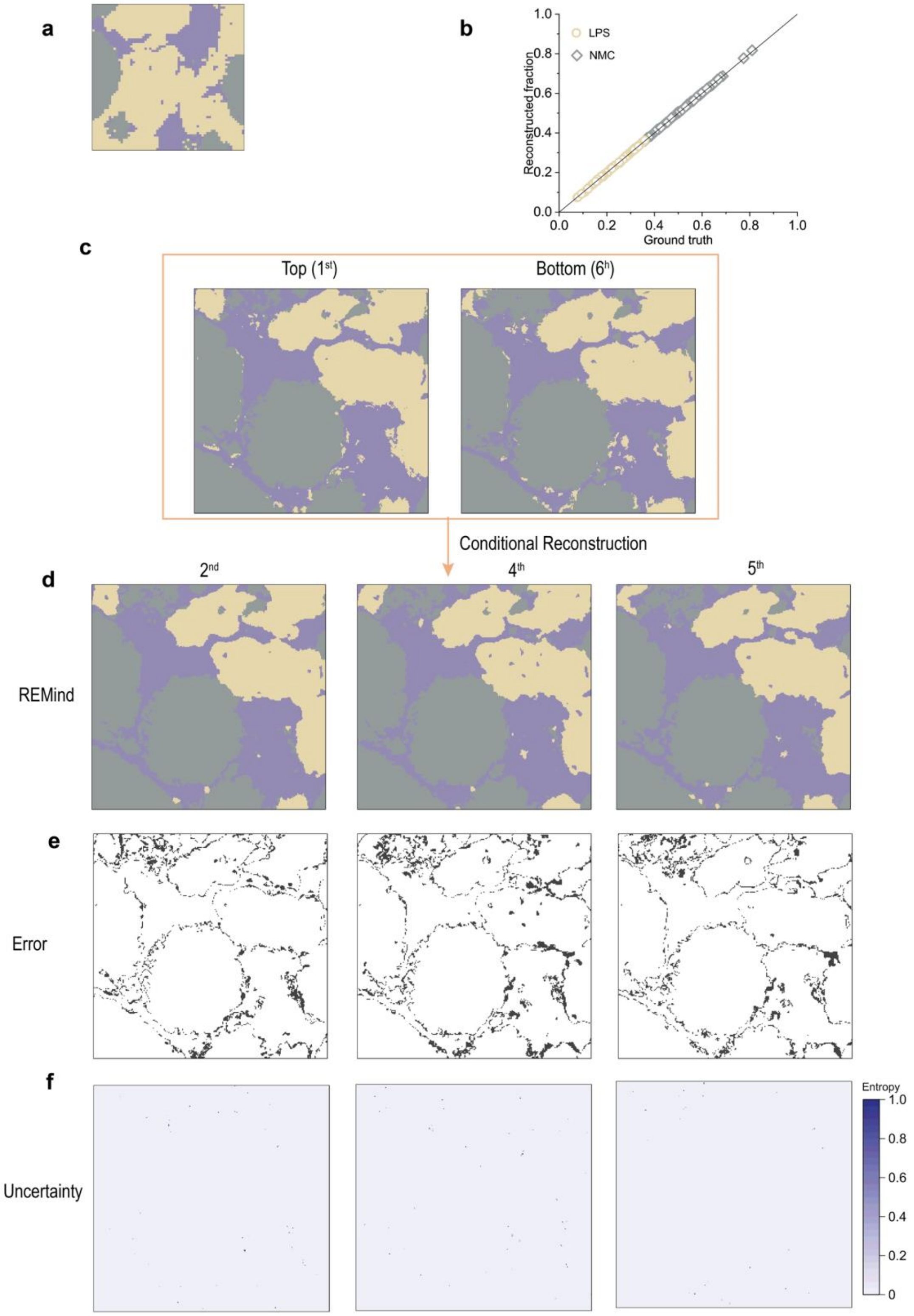

Figure S10 The performance of REMind on reconstructing the composite electrode of SSB. (a) Low-resolution input for the super-resolution at stage 1 of REMind under the factor 4 ×. (b) The reconstructed phase fraction across 100 super-resolution surfaces. (c) The input top and bottom surfaces of a given chunk to be reconstructed. (d) Reconstructed internal structures between the top and bottom surfaces. (e) The respective error distribution in each middle slice. (f) The distribution of entropy for uncertainty quantification.

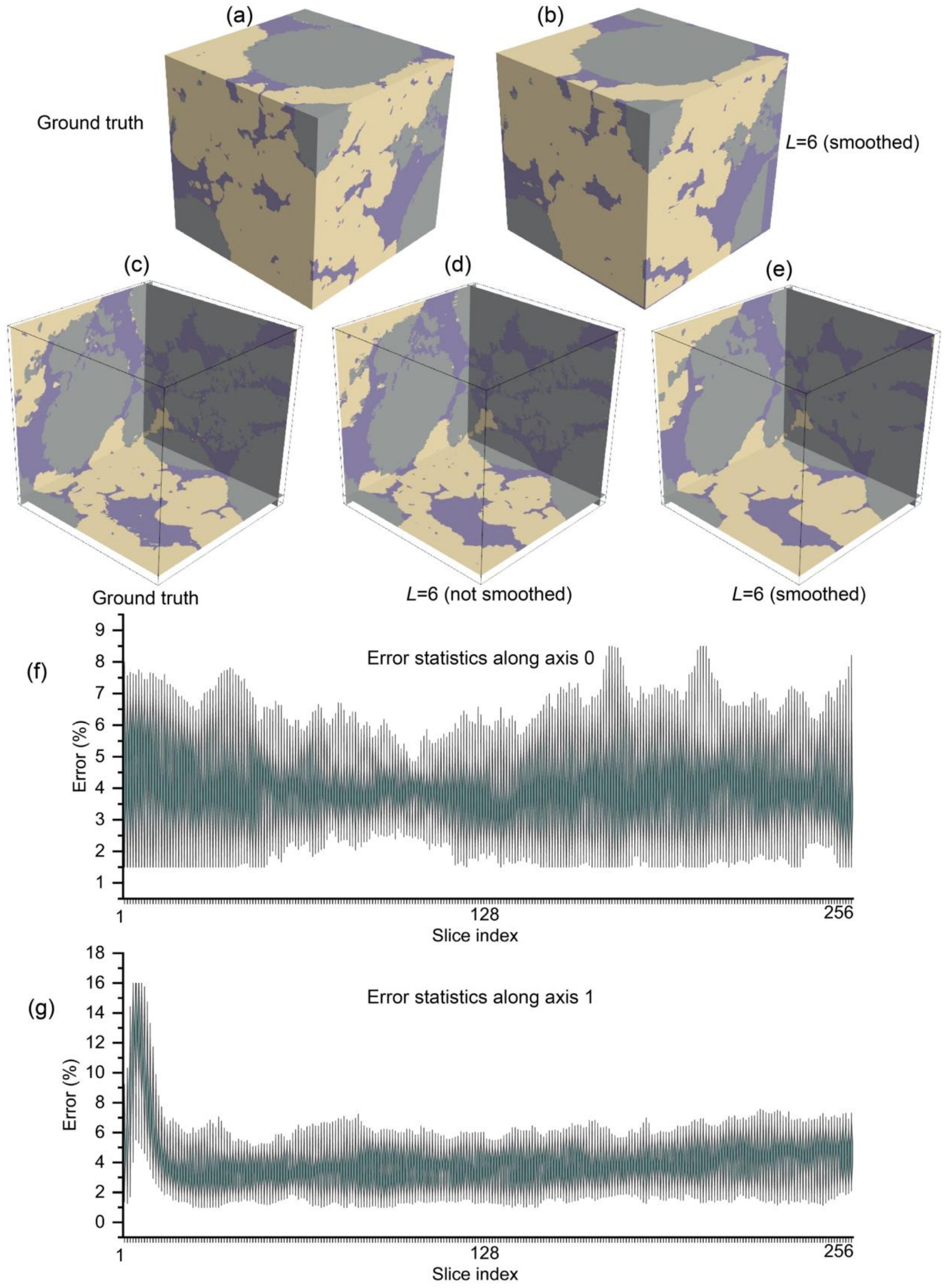

Figure S11 Cross-sectional error analysis and the performance of the smoothness algorithm of discontinuous patterns in the reconstructed SSB composite electrode samples. (a) Ground truth for the comparison; (b) The reconstructed sample, refined using the smoothness algorithm; (c-e) Cross-sectional slices of ground truth, the reconstructed sample without smoothness, and the reconstructed sample refined by the smoothness algorithm; (f) and (g) Error statistics across 20 reconstructed samples across axis 1 and axis 2 (the in-plane direction is denoted as axis 0).

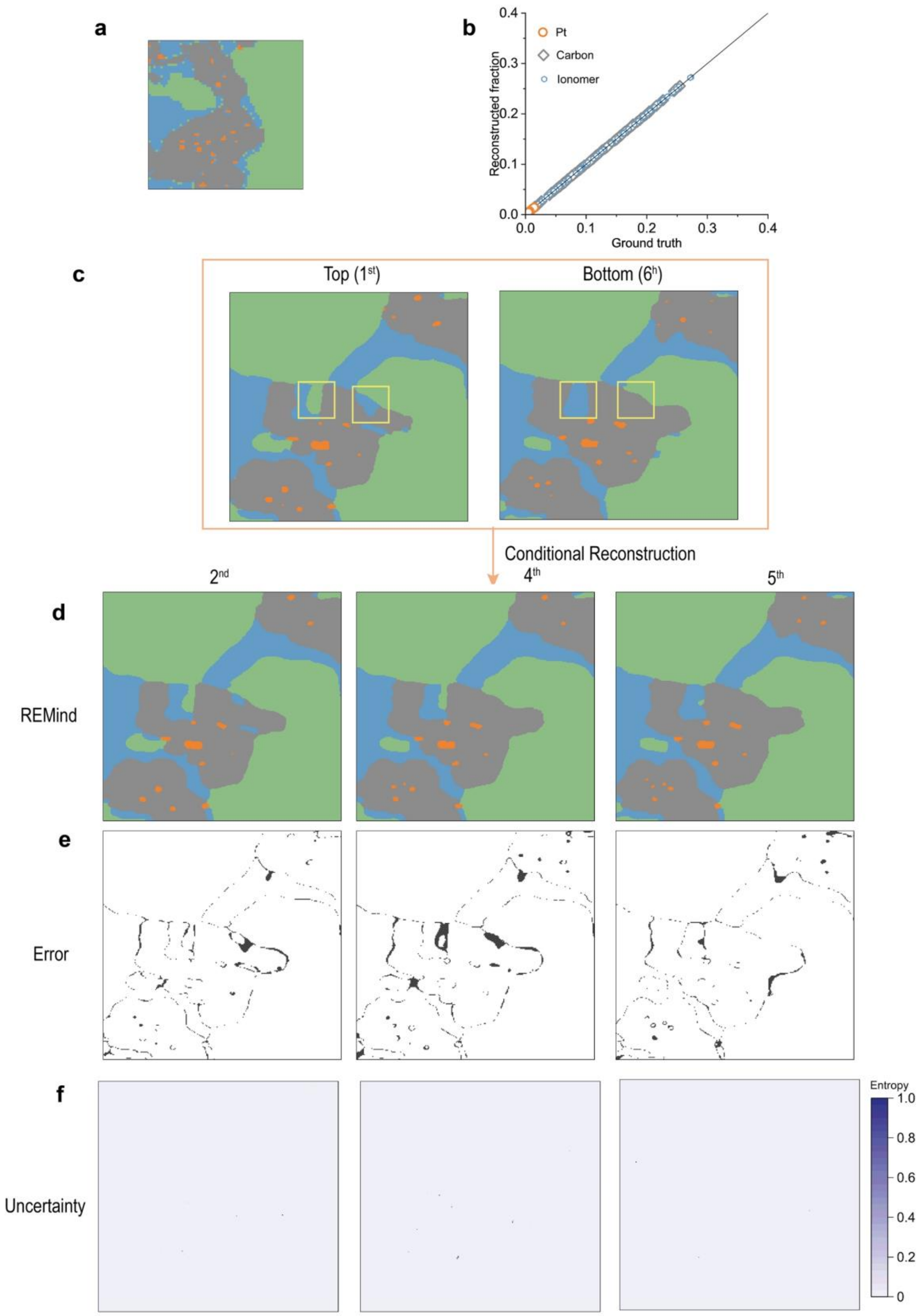

Figure S12 The performance of REMind on reconstructing the composite electrode of CLs. (a) Low-resolution input for the super-resolution at stage 1 of REMind under the factor 4 ×. (b) The reconstructed phase fraction across 100 super-resolution surfaces. (c) The input top and bottom surfaces of a given chunk to be reconstructed. (d) Reconstructed internal structures between the top and bottom surfaces. (e) The respective error distribution in each middle slice. (f) The distribution of entropy for uncertainty quantification.

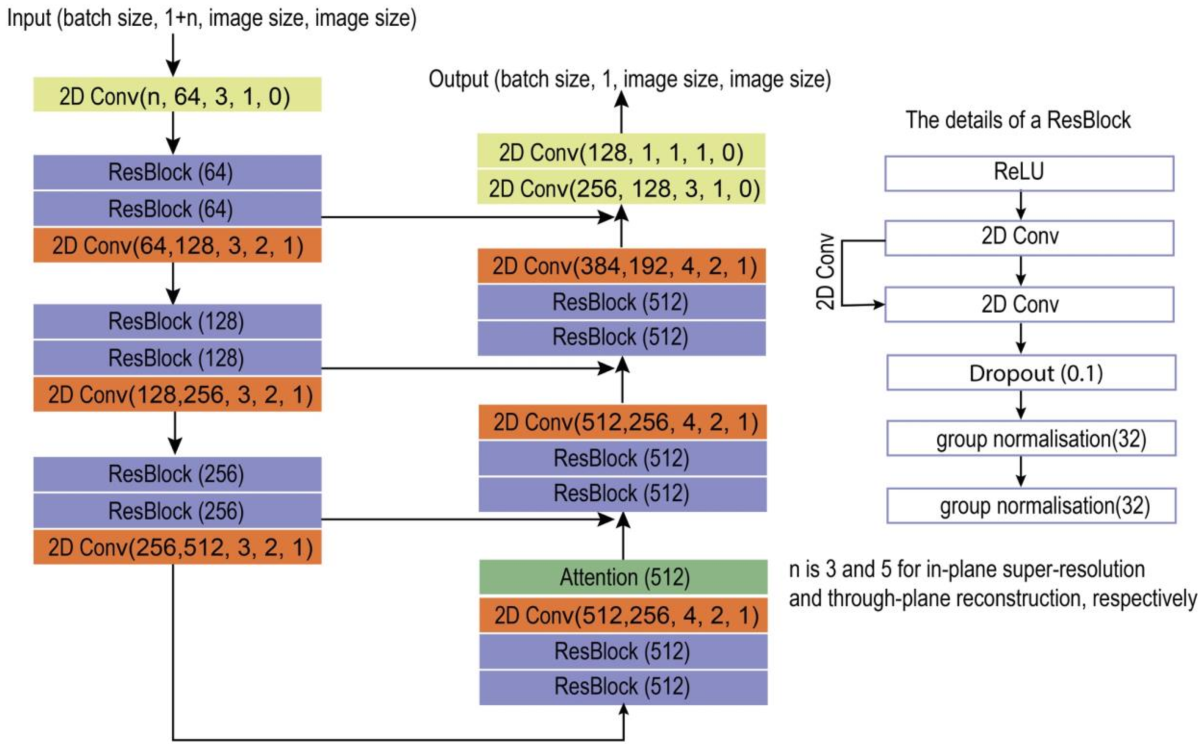

Figure S13 A U-net neural network architecture of REMind.

In Figure S13, we use the notion "2D Conv (C_{in}, C_{out}, k, s, p)" to describe the hyperparameters of each convolutional layer, where C_{in} represents the number of input channels, C_{out} represents the number of output channels, k refers to the kernel size, which defines the spatial dimensions of the convolutional filter (e.g., k=3 corresponds a 3×3 filter), s is the stride, which determines the step size with which the filter moves across the input. Larger stride values reduce the spatial dimensions of the output. p indicates the padding, referring to the number of pixels added to the input along the borders to control the spatial size of the output. For instance, "2D Conv(64, 128, 3, 2, 1)" represents a 2D convolutional layer with C_{in}=64 for input channels, C_{out}=128 for output channels, a kernel size of k=3, a stride of s=2, and padding of p=1. Additionally, we use the notion "Dropout(r)" to represent the dropout rate, which defines the probability of setting a neuron's output to zero during training. This regularization technique helps prevent overfitting by randomly deactivating a fraction of the neurons in each training iteration. A dropout rate of 0.1 is applied in all cases as designated by 'Dropout(0.1)'. Another important notion we used in Figure S12 is "Attention(d)" refers to an attention mechanism with a dimensionality of d for the query, key, and value representations. The attention mechanism allows the model to focus on relevant regions of the input by computing weighted contextual relationships, enhancing the capture of long-range dependencies. The attention layer in REMind is only active at the bottleneck layer of U-net. And "Attention(512)" indicates an attention layer where the query, key, and value vectors each have 512 channels.
